# Hec1-Dependent Cyclin B2 Stabilization Regulates the G2-M Transition and Early Prometaphase in Mouse Oocytes

**DOI:** 10.1016/j.devcel.2013.02.008

**Published:** 2013-04-15

**Authors:** Liming Gui, Hayden Homer

**Affiliations:** 1 Mammalian Oocyte and Embryo Research Laboratory, Cell and Developmental Biology, UCL, London WC1E 6BT, UK; 2 Reproductive Medicine Unit, Institute for Women’s Health, UCLH Elizabeth Garrett Anderson Wing, London NW1 2BU, UK

## Abstract

The functions of the Ndc80/Hec1 subunit of the highly conserved Ndc80 kinetochore complex are normally restricted to M phase when it exerts a pivotal kinetochore-based role. Here, we find that in mouse oocytes, depletion of Hec1 severely compromises the G2-M transition because of impaired activation of cyclin-dependent kinase 1 (Cdk1). Unexpectedly, impaired M phase entry is due to instability of the Cdk1-activating subunit, cyclin B2, which cannot be covered by cyclin B1. Hec1 protects cyclin B2 from destruction by the Cdh1-activated anaphase-promoting complex (APC^Cdh1^) and remains important for cyclin B2 stabilization during early M phase, required for the initial stages of acentrosomal spindle assembly. By late M phase, however, Hec1 and cyclin B2 become uncoupled, and although Hec1 remains stable, APC^Cdc20^ triggers cyclin B2 destruction. These data identify another dimension to Hec1 function centered on M phase entry and early prometaphase progression and challenge the view that cyclin B2 is completely dispensable in mammals.

## Graphical abstract

**Graphical Abstract:**

**Figure:**
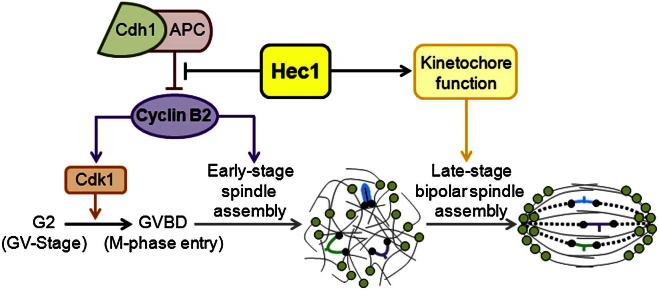

**Highlights:**

► Hec1 stabilizes cyclin B2 against APC^Cdh1^-mediated destruction in mouse oocytes ► Hec1, through cyclin B2, is important for Cdk1 activation at the G2-M transition ► Hec1-dependent cyclin B2 stabilization is required for early-stage spindle assembly ► Hec1’s kinetochore-based role promotes late-stage spindle assembly

## Teaser

Hec1 is a subunit of the Ndc80 kinetochore complex best known for linking kinetochores to microtubules during M phase. Gui and Homer find that in mouse oocytes, Hec1 plays a kinetochore-independent role in stabilizing cyclin B2, which promotes the G2-M transition and early-stage acentrosomal spindle assembly during meiosis I.

## Introduction

The Ndc80 complex, comprised of the Hec1, Nuf2, Spc24, and Spc25 subunits, is a highly conserved kinetochore component (Ciferri et al., 2007). The N-terminal region of Hec1 is important for mediating microtubule binding and for spindle assembly checkpoint (SAC) function by regulating the kinetochore localization of SAC components, such as Mad1 and Mad2 (Ciferri et al., 2007; DeLuca et al., 2003; Hori et al., 2003; Martin-Lluesma et al., 2002). Although its kinetochore-based roles have taken center stage, Hec1 also has at least one other function involving centrosome-mediated microtubule nucleation through an interaction with Hice1 (Wu et al., 2009). Indeed, Hec1 function could be even more diverse as the C-terminal portion of Hec1 interacts with a range of cellular regulators and in vitro assays raise the possibility that one such interaction could serve to modulate proteolysis of pivotal cell-cycle regulators, such as cyclins (Chen et al., 1997). Significantly, however, it is not yet known whether Hec1 exerts any physiologically relevant roles beyond either the chromosome segregation machinery or M phase.

It is widely held that cyclin B2 (encoded by *CCNB2*) is dispensable in mammals (Brandeis et al., 1998) as, during mitosis, its loss can be fully covered by other cyclins, such as cyclin B1 and cyclin A2 (Bellanger et al., 2007; Gong et al., 2007). Significantly, a prominent feature of mammalian oocytes not shared with mitosis is Cdh1-activated APC activity (APC^Cdh1^) during prophase and early prometaphase (Homer et al., 2009; Homer, 2011; Reis et al., 2006, 2007), which, by severely restraining cyclin accumulation, could limit the capacity of cyclins to cover for one another.

Although *HEC1* and *CCNB2* RNA expression have previously been documented in mouse oocytes (Chapman and Wolgemuth, 1993; Ledan et al., 2001; Sun et al., 2011), here we detail their protein expression and, importantly, determine how measured reductions in their endogenous protein levels affect meiosis I (MI). This led us to identify a Hec1-cyclin B2 regulatory pairing that not only extends Hec1’s function beyond M phase but also defines an important role for cyclin B2 in mammals.

## Results

**Figure 1:**
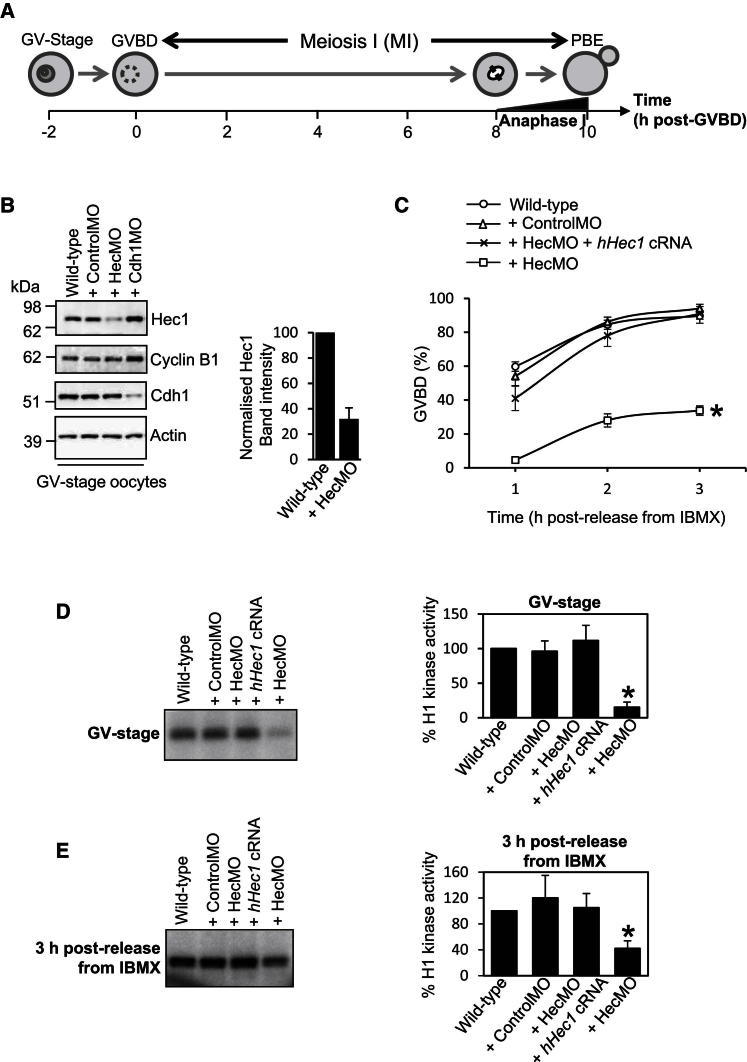
Hec1 Depletion Impairs GVBD and Cdk1 Activity (A) Schematic of MI in mouse oocytes. First polar body extrusion (PBE) marks exit from MI. (B) Immunoblot of Hec1 (∼79 kDa) (Diaz-Rodríguez et al., 2008), cyclin B1 and Cdh1 in wild-type, mock-depleted (+ ControlMO), Hec1-depleted (+ HecMO), and Cdh1-depleted (+ Cdh1MO) GV-stage oocytes (50 oocytes per sample). Hec1 band intensities from four separate experiments were normalized to values found in wild-type oocytes. (C) GVBD rates at 1, 2, and 3 hr following washout from IBMX for Hec1-depleted (n = 574), mock-depleted (n = 147), and Hec1-depleted oocytes coexpressing hHec1 from injected cRNA (+ HecMO + *hHec1* cRNA; n = 86). (D and E) Histone H1 kinase activity at either the GV-stage (D) or 3 hr following release from IBMX (E). Mean kinase activities from three separate experiments were normalized to activity in wild-type oocytes. Data are mean ± SEM. ^∗^p < 0.0001.

Mammalian oocytes experience a protracted G2-prophase arrest characterized by the presence of an intact germinal vesicle (GV; Figure 1A), the term used for the oocyte’s large and easily identifiable nucleus. Notably, G2 arrest can be efficiently maintained in vitro using drugs, such as 1-isobutyl 3-methylxanthine (IBMX) (Homer et al., 2009; Marangos et al., 2007; Marangos and Carroll, 2008), following washout from which, oocytes spontaneously undergo GV breakdown (GVBD; Figure 1A), signifying entry into M phase. Thus, the ability to easily monitor and to reversibly modulate the events surrounding the G2-M transition make mouse oocytes a powerful model for studying the regulation of this fundamental cell-cycle transition.

### Hec1 Depletion Impairs GVBD and Cdk1 Activity Independent of Cyclin B1 or Cdh1

For depleting Hec1 in mouse oocytes, we used a morpholino antisense approach we used previously (Gui and Homer, 2012; Homer et al., 2005b, 2009). We found that microinjection of a morpholino designed against *mHEC1* (designated HecMO) into GV-stage oocytes followed by a 24 hr incubation in IBMX produced 60%–70% depletion of Hec1 (Figure 1B). In contrast, neither mock depletion nor depletion of Cdh1 using a well-characterized *mCDH1*-targeting morpholino (Cdh1MO) (Homer et al., 2009; Reis et al., 2006) reduced Hec1 levels (Figure 1B).

Unexpectedly, by 3 hr following release from IBMX, only ∼34% of Hec1-depleted oocytes underwent GVBD compared with 80%–90% GVBD rates in wild-type oocytes (Figure 1C). Furthermore, using histone H1 kinase assays, we found that Cdk1 activity in Hec1-depleted oocytes was less than 20% of wild-type levels at the GV stage and attained less than half the activity of wild-type oocytes by 3 hr following release from IBMX (Figures 1D and 1E). In contrast, neither Cdk1 activity nor GVBD rates were affected by mock depletion, and both could be restored in Hec1-depleted oocytes by coexpressing human Hec1 (hHec1) from exogenous cRNA (Figures 1C–1E).

Thus far, the only Cdk1-activating cyclin with a proven role at the G2-M boundary of MI in mouse oocytes is cyclin B1, whose APC^Cdh1^-mediated destruction is indispensable for preventing unscheduled Cdk1 activation during G2 arrest (Reis et al., 2006). Consequently, alterations in cyclin B1 and/or Cdh1 levels characterize many conditions that perturb Cdk1 activity and entry into M phase (Homer et al., 2009; Marangos et al., 2007; Marangos and Carroll, 2008; Schindler and Schultz, 2009). Significantly, however, neither Cdh1 nor cyclin B1 levels—the latter detected using an antibody that we and others have found to detect cyclin B1 (∼60 kDa) in mouse oocytes (Holt et al., 2010; Homer et al., 2009; Marangos and Carroll, 2008; Oh et al., 2011; Reis et al., 2007)—were altered in Hec1-depleted oocytes (Figure 1B). Surprisingly, therefore, Hec1 depletion led to reductions in GVBD and Cdk1 activity without impacting the canonical APC^Cdh1^-cyclin B1 pathway.

### Impaired GVBD after Hec1 Depletion Is Due to Reduced Cyclin B2 Levels that Are Not Covered by Cyclin B1

**Figure 2:**
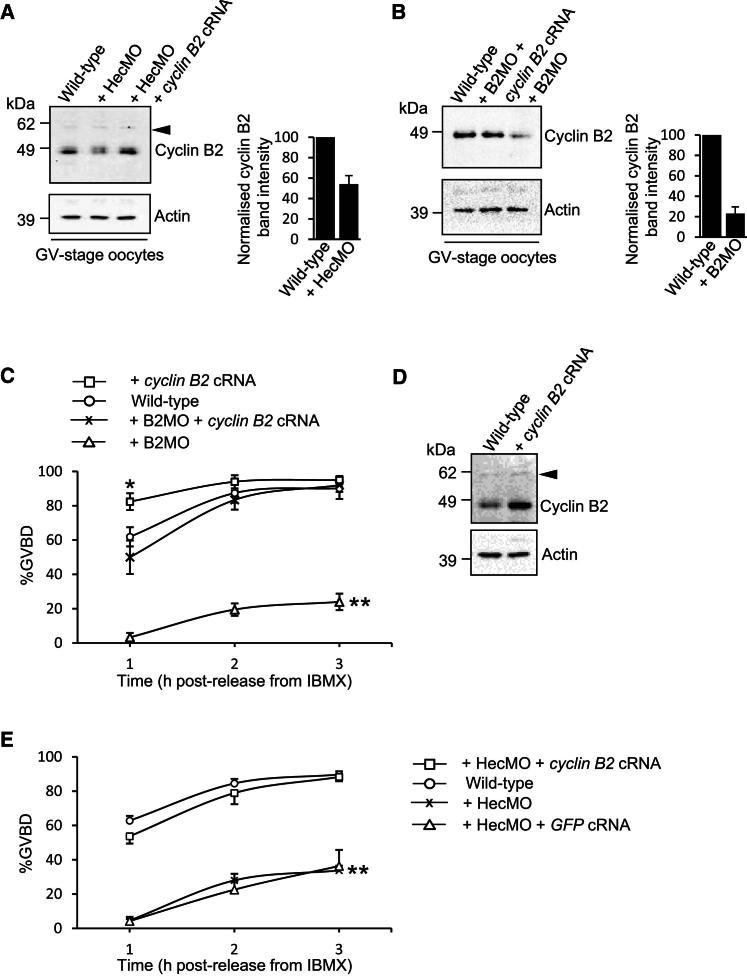
Hec1-Dependent Stabilization of Cyclin B2 Is Required for GVBD (A and B) Immunoblot of cyclin B2 (∼45 kDa) in Hec1-depleted oocytes and Hec1-depleted oocytes coinjected with *cyclin B2* cRNA (+ HecMO + *cyclin B2* cRNA) (A), as well as in cyclin-B2-depleted oocytes (+ B2MO) and in cyclin-B2-depleted oocytes coinjected with *cyclin B2* cRNA (+ B2MO + *cyclin B2* cRNA) (B). (C and E) GVBD rates at 1, 2, and 3 hr following washout from IBMX for wild-type oocytes, cyclin-B2-depleted oocytes (n = 141), + B2MO + *cyclin B2* cRNA oocytes (n = 56), and oocytes overexpressing cyclin B2 (+ *cyclin B2* cRNA; n = 76) (C), as well as Hec1-depleted, + HecMO + *cyclin B2* cRNA oocytes (n = 104) and + HecMO + *GFP* cRNA oocytes (n = 73) (E). (D) Immunoblot showing increased cyclin B2 following microinjection of *cyclin B2* cRNA. Black arrowheads (A and D) mark the predicted position for cyclin B1 (see Figure 1B), where a faint band is occasionally detected. Data are mean ± SEM. ^∗^p = 0.0068 at 1 hr time point; ^∗∗^p < 0.0001 at all three time points. See also Figure S1.

We turned our attention to the other major Cdk1-activating B-type cyclin in mammals, cyclin B2. We used an antibody that produced a strong signal for a band that migrated to cyclin B2’s predicted position (∼45 kDa), distinct from the position of cyclin B1 (∼60 kDa), where we inconsistently detected a much weaker signal (see Figures 2A, 2B, and 2D). As confirmation that the ∼45 kDa band did indeed represent cyclin B2, this band’s intensity was markedly reduced following injection of a *CCNB2*-targeting morpholino oligonucleotide (designated B2MO; see Figure 2B), restored by coexpressing cyclin B2 from exogenous cRNA (see Figure 2B) and increased when cyclin B2 cRNA was microinjected into wild-type oocytes (see Figure 2D). Significantly, none of these interventions had any discernible impact on the slower migrating band (see Figures 2A and 2D). We therefore conclude that the ∼45 kDa band represents cyclin B2, thereby enabling us to confidently monitor changes in its levels.

**Figure 3:**
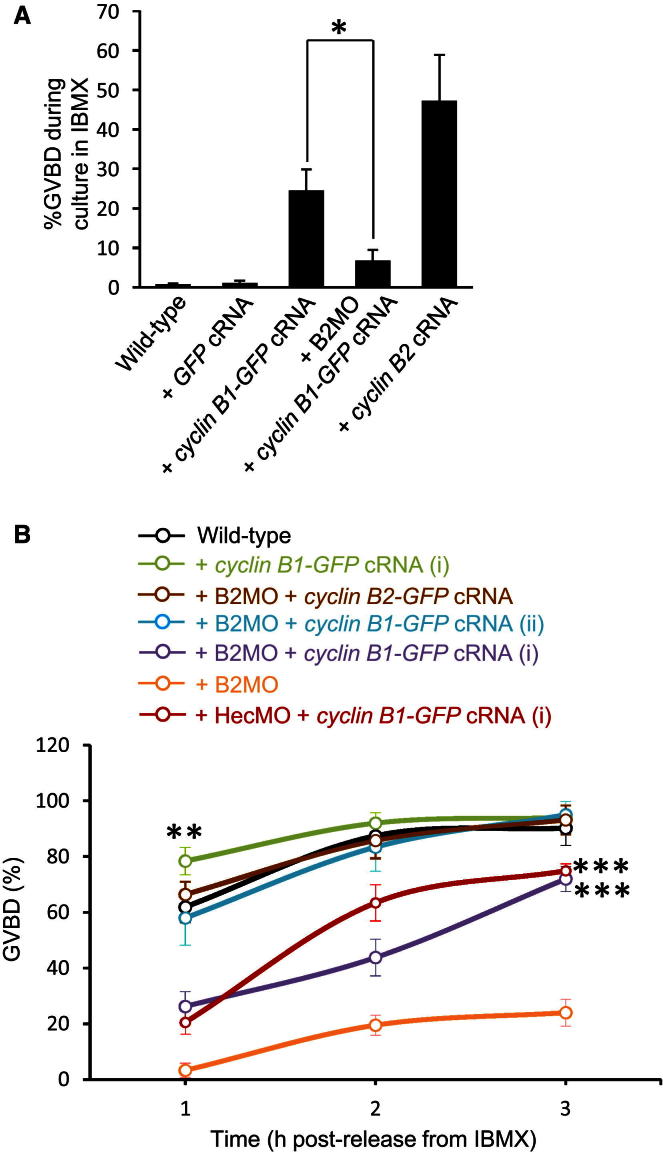
The Ability of Cyclin B1-GFP to Promote GVBD Is Significantly Impaired on a Cyclin B2 Knockdown Background (A) Cyclin B2 depletion impairs the ability of cyclin B1-GFP overexpression to induce escape from IBMX-mediated G2-prophase arrest. We microinjected wild-type oocytes with *GFP* cRNA (+ *GFP* cRNA; n = 12), *cyclin B1-GFP* cRNA (+ *cyclin B1-GFP* cRNA; n = 23), or *cyclin B2* cRNA (+ *cyclin B2* cRNA; n = 15) and microinjected cyclin-B2-depleted oocytes with *cyclin B1-GFP* cRNA (+ B2MO + *cyclin B1-GFP* cRNA; n = 25). Oocytes were then maintained in culture medium containing 50 μM IBMX along with control uninjected oocytes and scored for spontaneous GVBD rates 24 hr postinjection. Note that GVBD rates in oocytes overexpressing cyclin B2 are roughly double that of oocytes overexpressing cyclin B1-GFP. ^∗^p = 0.0036. (B) Following cyclin B2 depletion, the ability of cyclin B1 overexpression to promote M phase entry is compromised and less efficient than cyclin B2 overexpression. *Cyclin B1-GFP* cRNA was microinjected into wild-type oocytes (+ *cyclin B1-GFP* cRNA; n = 32), cyclin B2 deleted oocytes (+ B2MO + *cyclin B1-GFP* cRNA; n = 41), or Hec1 depleted oocytes (+ HecMO + *cyclin B1-GFP* cRNA; n = 38), whereas *cyclin B2-GFP* cRNA was injected into cyclin-B2-depleted oocytes (+ B2MO + *cyclin B2-GFP* cRNA; n = 34). Following microinjection of *cyclin B1-GFP* cRNA, oocytes were maintained for either 2 hr (denoted by i) or 6 hr (denoted by ii) in 200 μM IBMX, producing about a 2-fold difference in cyclin B1-GFP expression as explained in greater detail in Figure S2. Following microinjection of *cyclin B2-GFP* cRNA, oocytes were maintained for 2 hr in 200 μM IBMX. Oocytes were then washed into IBMX-free culture medium and scored for GVBD at hourly intervals along with uninjected wild-type oocytes and cyclin-B2-depleted oocytes (+ B2MO). Data are mean ± SEM. ^∗∗^p = 0.031 (versus wild-type) for 1 hr time point; ^∗∗∗^p < 0.05 (versus wild-type) for all three time points. See also Figure S2.

We found that, in marked contrast to cyclin B1, levels of cyclin B2 were roughly halved following Hec1 depletion (Figure 2A; Figures S1A and S1B available online). Reduced cyclin B2 was specifically related to reduced Hec1 as coexpression of hHec1 from exogenous cRNA in Hec1-depleted oocytes restored cyclin B2 levels (Figures S1C and S1D). This indicated that Hec1 might modulate Cdk1 activity through cyclin B2. To explore this further, we next examined whether depleting cyclin B2 would impact M phase entry. By microinjecting B2MO, we were able to induce 70%–80% cyclin B2 knockdown in GV-stage oocytes following 24 hr of incubation in IBMX (Figure 2B). Strikingly, in oocytes depleted of cyclin B2 by B2MO (hereafter cyclin-B2-depleted oocytes), GVBD rates only attained ∼20% (Figure 2C), mirroring the impairment observed after Hec1 depletion. We note that B2MO incurred a somewhat more severe GVBD defect than HecMO, consistent with a more severe cyclin B2 depletion induced by B2MO. Furthermore, we found that overexpression of cyclin B2 from exogenous cRNA (Figure 2D) not only accelerated GVBD (Figure 2C) but also led to 40%–50% spontaneous GVBD during culture in IBMX (Figure 3A). Entirely consistent with our findings, markedly increased GVBD rates occurred following microinjection of polyadenylated *cyclin B2* cRNA (Ledan et al., 2001). We note, however, that the same study did not observe any effect following microinjection of a *CCNB2*-targeting antisense RNA (Ledan et al., 2001). The latter may have been due to less severe protein knockdown than we achieved here as antisense RNA-injected oocytes were maintained at the GV stage for only 4–5 hr (Ledan et al., 2001), whereas we found that a 24 hr incubation in IBMX post-B2MO injection was required for inducing substantial depletion.

Comparable cyclin B1 levels between Hec1-depleted and wild-type oocytes (Figure 1B) suggested that cyclin B1 could not readily cover the G2-M defect arising from reductions in cyclin B2. In line with this, overexpression of a GFP-tagged cyclin B1 construct (cyclin B1-GFP)—which is known to promote GVBD (Holt et al., 2010; Ledan et al., 2001; Reis et al., 2006)—in cyclin-B2-depleted oocytes induced 3–4 times lower rates of spontaneous GVBD during culture in IBMX than did cyclin B1-GFP overexpression in wild-type oocytes (Figure 3A). Furthermore, levels of cyclin B1-GFP overexpression that were sufficient to accelerate GVBD in wild-type oocytes only partially rescued GVBD following either Hec1 or cyclin B2 depletion, the full restoration of which required ∼2-fold higher cyclin B1-GFP expression (Figures 3B, S2A, S2C, and S2D). In order to directly compare the ability of cyclin B1 and cyclin B2 to reverse the defect in GVBD after cyclin B2 depletion, we used a GFP-tagged cyclin B2 construct. We found that cyclin B2-GFP was capable of fully restoring GVBD in cyclin-B2-depleted oocytes (Figure 3B). Highly significantly, using GFP fluorescence to estimate protein expression, the levels of cyclin B2-GFP that were capable of fully restoring GVBD were lower than those at which cyclin B1-GFP could only partially restore GVBD (Figure 3B; Figures S2B–S2D). Overall, these data show that, unlike mitosis (Bellanger et al., 2007), cyclin B1 could not readily compensate for cyclin B2 loss in oocytes. In contrast, replenishing endogenous cyclin B2 to wild-type levels in either Hec1-depleted or cyclin-B2-depleted oocytes (see Figures 2A and 2B) fully restored GVBD (Figures 2C and 2E). Thus, Hec1 is required to stabilize cyclin B2, which in turn plays an indispensable role in Cdk1 activation required for the G2-M transition.

### Hec1-Dependent Cyclin B2 Stabilization during Early M Phase Is Important for Early-Stage Spindle Assembly

**Figure 4:**
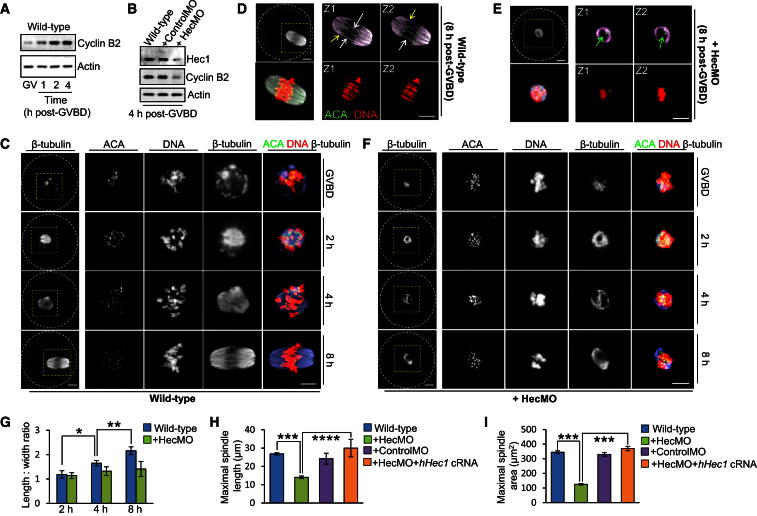
Hec1 Stabilizes Cyclin B2 during Early Prometaphase and Is Required for Early-Stage Spindle Assembly (A) Immunoblot of cyclin B2 during early prometaphase in wild-type oocytes. (B) Immunoblot of Hec1 and cyclin B2 in wild-type oocytes, mock-depleted (+ ControlMO), and Hec1-depleted (+HecMO) oocytes. Fifty oocytes per sample. (C–F) Confocal images of wild-type (C and D) and Hec1-depleted (E and F) oocytes immunostained for DNA, kinetochores (ACA), and microtubules (β-tubulin) at the times shown post-GVBD. Z1 and Z2 (D and E) represent individual confocal Z sections. Scale bars, 10 μm. (G) Graph showing length: width ratios for wild-type and Hec1-depleted oocytes at 2 hr (n = 18), 4 hr (n = 12), and 8 hr (n = 15) post-GVBD. (H and I) Maximal spindle lengths (H) and spindle areas (I) in wild-type oocytes (n = 32), Hec1-depleted oocytes (n = 26), mock-depleted oocytes (n = 15), and Hec1-depleted oocytes coexpressing hHec1 (+ HecMO + *hHec1* cRNA; n = 16). Data are mean ± SEM. ^∗^p = 0.033; ^∗∗^p = 0.0196. ^∗∗∗^p < 0.0001; ^∗∗∗∗^p = 0.0011. See also Figure S3.

We found that following GVBD, cyclin B2 underwent markedly increased synthesis during early prometaphase in wild-type oocytes (Figure 4A). Surprisingly, however, following Hec1 depletion, cyclin B2 levels remained reduced even after 4 hr of M phase (Figure 4B), indicating that Hec1 remained an independent determinant of cyclin B2 levels in early prometaphase that could not be compensated for by increased cyclin B2 synthesis. In keeping with an exquisite sensitivity to Hec1 levels, we could not readily overexpress cyclin B2 on a Hec1 knockdown background (compare Figures 2A and 2D).

In order to further characterize the meiotic defect after Hec1 depletion, we next analyzed spindle assembly. Notably, unlike mitosis, in which spindle bipolarity is predefined by a pair of centrosomes, in oocytes, spindle assembly is brought about by microtubule nucleation from multiple microtubule organizing centers (MTOCs) (Schuh and Ellenberg, 2007). In wild-type oocytes, the earliest stage of spindle assembly shortly after GVBD is characterized by a spherically shaped spindle with a low-density interior that is occupied by clumped chromosomes (Figure 4C; GVBD). Subsequently, the spindle becomes molded over 4–8 hr into a barrel-shaped bipolar structure (Figures 4C and 4D), coinciding with sorting of MTOCs into two distinct poles (Breuer et al., 2010; Gui and Homer, 2012; Kolano et al., 2012). The fully formed bipolar spindle is markedly different from the earliest spindle form and is characterized by a highly organized array of antiparallel running microtubule bundles comprised of cold-stable kinetochore microtubules (or K-fibers) alternating with interpolar microtubule bundles (Figures 4D and S3A).

Along with spindle remodeling, chromosome morphology is also subject to dramatic changes during MI. The first overt change is that densely clumped recombined homologous chromosomes (termed bivalents) become discernible as individual structures (termed individualization; Figures 4C, S3C, and S3D). Following individualization, and while the spindle is being molded into a bipolar form, bivalents gradually become “stretched” from an initially compact structure with juxtaposed kinetochores (hereafter compact bivalent) to an extended structure with kinetochores facing in opposite directions (hereafter extended bivalent) (Figures S3C–S3I) (Gui and Homer, 2012; Kitajima et al., 2011).

Strikingly, after Hec1 depletion, we found that spindles at 8 hr post-GVBD were characterized by prominent “windows” that surrounded densely clumped chromosomes, features that were readily apparent on individual confocal Z sections (Figure 4E). This morphology was more reminiscent of the earliest stage of wild-type spindle assembly (see Figure 4C; GVBD) rather than the typical bipolar morphology that was ordinarily present by late MI (compare Figures 4D and 4E) and was present in Hec1-depleted oocytes throughout MI pointing to a chronic impairment of spindle assembly (Figure 4F). In keeping with early-stage stagnation, and in sharp contrast with the marked spindle elongation that accompanied bipolarization in wild-type oocytes, after Hec1 depletion spindle lengths and length-to-width ratios showed only modest increases, resulting in significantly reduced spindle lengths and areas (Figures 4G–4I). Furthermore, in contrast to wild-type bipolar spindles, in which K-fibers were a prominent feature by late MI (Figure S3A) (Gui and Homer, 2012; Homer et al., 2009), Hec1-depleted spindles were markedly deficient in K-fibers (Figure S3B), consistent with Hec1’s known role in stabilizing kinetochore-microtubule attachments (DeLuca et al., 2005). Spindle defects were specific to Hec1 depletion as spindle assembly and individualization were not impaired in either mock-depleted oocytes or in Hec1-depleted oocytes coexpressing hHec1 (Figures S3J–S3L). Thus, spindle assembly arrested at an early stage after Hec1 depletion.

**Figure 5:**
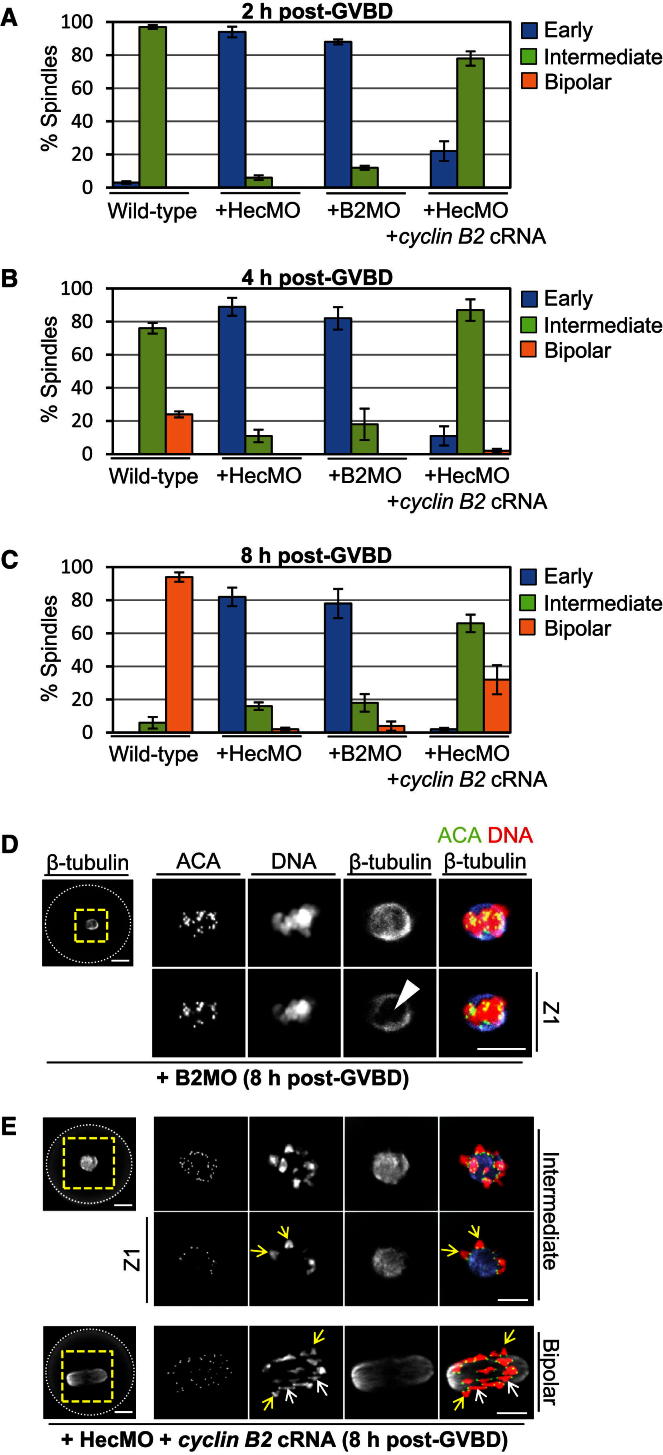
Compromised Early-Stage Spindle Assembly after Hec1 Depletion Is Related to Cyclin B2 Instability (A–C) Confocal analyses of immunostained oocytes (see D and E) were used to determine the proportions of oocytes with bipolar spindles (defined as having a clear bipolar appearance and a length: width ratio > 1.8; see Figure 4D), early spindles (defined as spindles with windows containing clumped chromosomes, see D), and intermediate spindles (on the basis that chromosomes have individualized and spindles lack windows but bipolarization is incomplete, see E). Data are mean ± SEM. (D and E) Confocal immunostained images depict the early-stage morphology typical of cyclin-B2-depleted oocytes (D) as well as intermediate and bipolar spindle morphologies in Hec1-depleted oocytes coexpressing cyclin B2 (E). Note the presence of large windows readily apparent on individual confocal Z sections (D, white arrowhead, Z1) occupied by chromosomal clusters associated with the early morphology. In contrast, in the intermediate morphology, spindle windows are not detectable on individual confocal Z sections (E, Intermediate, Z1) and individual chromosomes are clearly discernible (E, Intermediate, yellow arrows, Z1). For comparison, a bipolar spindle morphology is shown. Note that all bivalents are of a compact configuration in the intermediate spindle, whereas in the bipolar spindle many bivalents are extended (white arrows) with smaller numbers of compact bivalents (yellow arrows). Note also that oocytes were fixed at 8 hr post-GVBD when wild-type oocytes have almost always completed bipolar spindle assembly (see C). Panels to the left are whole-oocyte images in the β-tubulin channel with the dashed white circles outlining the oocyte, whereas panels to the right are magnified images of the region enclosed by the dashed yellow squares. Scale bars, 10 μm.

Strikingly, we found that cyclin B2 depletion also severely compromised spindle assembly and significantly resulted in a morphology indistinguishable from that in Hec1-depleted oocytes (Figure 5D). Added to this, cyclin B2 coexpression in Hec1-depleted oocytes enabled spindle assembly to advance beyond the early stages in over 95% of cases (Figures 5A–5C), as chromosomes were able to individualize and large windows in spindles were no longer prominent (Figure 5E). Thus, Hec1-dependent cyclin B2 stabilization is important during early prometaphase for the initial stages of spindle assembly required for chromosomal individualization.

### 9A-Hec1 and CENP-E Depletion Predominantly Impair the Latter Stages of Acentrosomal Spindle Assembly

We observed that although a very small proportion of oocytes (∼2%) retained a rudimentary morphology when cyclin B2 was coexpressed in Hec1-depleted oocytes, a far more severe defect was incurred during later-stage bipolarization; although cyclin B2 coexpression in Hec1-depleted oocytes enabled spindle assembly to progress beyond the early stages in over 95% of oocytes, ∼68% of such oocytes could not complete the assembly of proper bipolar spindles (Figures 5A–5C). Altogether, this pointed to a cyclin-B2-independent function for Hec1 that was important for acentrosomal spindle assembly, especially during the latter stages. This led us to examine whether Hec1’s more conventional kinetochore-related function might be important for acentrosomal spindle assembly.

The efficient formation of stable attachments between kinetochores and microtubules is critically dependent upon the N-terminal tail of Hec1, the phosphorylation status of which is a major determinant of microtubule attachment affinity (Ciferri et al., 2008; DeLuca et al., 2006; Guimaraes et al., 2008; Miller et al., 2008; Sundin et al., 2011; Wei et al., 2007; Welburn et al., 2010). Consequently, a deplete-and-rescue approach that effectively replaces endogenous Hec1 with a nonphosphorylatable Hec1 N-terminal tail domain mutant (9A-Hec1) significantly impairs Hec1’s kinetochore function in mitosis (DeLuca et al., 2011; Guimaraes et al., 2008; Sundin et al., 2011). In order to explore a potential kinetochore-based role for Hec1 in oocytes therefore, we employed 9A-Hec1 in a similar deplete-and-rescue approach.

**Figure 6:**
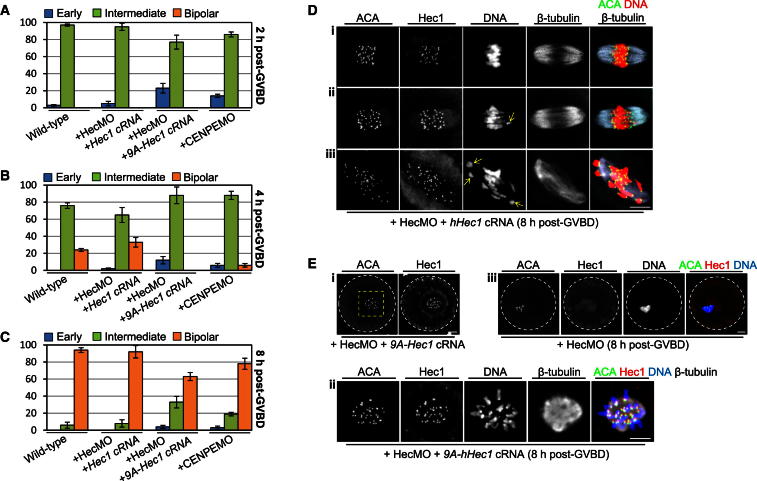
Impaired Kinetochore Function Predominantly Compromises Later Stages of Spindle Assembly (A–C) Characterization of spindle morphology using the criteria set out in Figure 5 in wild-type oocytes, Hec1-depleted oocytes coexpressing either hHec1 (HecMO + *hHec1* cRNA; n = 46) or 9A-Hec1 (HecMO + *9A-Hec1* cRNA; n = 52) and CENP-E-depleted oocytes (+ CENPEMO; n = 48). Data are mean ± SEM. (D and E) Confocal immunostained images depict intact bipolar spindle assembly typical of Hec1-depleted oocytes rescued with hHec1 (D) and an intermediate morphology in a Hec1-depleted oocyte coexpressing 9A-Hec1 (E, i and ii). For comparison, a Hec1-depleted oocyte is shown in which Hec1 is largely undetectable at kinetochores, indicating that the kinetochore signal in Hec1-depleted oocytes coexpressing 9A-Hec1 represents 9A-Hec1. Note also the clumped chromosomes after Hec1 depletion (E, iii) and that although chromosomes individualize with 9A-Hec1, kinetochore reorientation is impaired producing multiple compact bivalents (E, ii), whereas with wild-type hHec1 the majority of oocytes display all extended bivalents (37 of 54; D, i) with only a minority of oocytes displaying either a single compact bivalent (yellow arrow; 10 of 54; D, ii) or 2–4 compact bivalents (7 of 54; D, iii). Panels in (E, ii) are magnified images of the region enclosed by the dashed yellow square in (E, i). Scale bars, 10 μm. See also Figure S4.

We examined the effect of either wild-type hHec1 or 9A-Hec1 (expressed from microinjected cRNAs) on spindle assembly in Hec1-depleted oocytes. As predicted, coexpression of hHec1 fully restored spindle assembly in Hec1-depleted oocytes (Figures 6A–6D; see also Figure S3L). In stark contrast, although 9A-Hec1 markedly improved spindle assembly after Hec1 depletion, significant defects remained, especially involving later-stage bipolarization (Figures 6A–6C and 6E). We attribute such defects with 9A-Hec1 to disrupted kinetochore function as 9A-Hec1 localized strongly to kinetochores and markedly disrupted kinetochore reorientation (Figure 6E). In contrast, only minimal defects in kinetochore reorientation were observed following coexpression of wild-type hHec1, which also localized to kinetochores (Figure 6D). Furthermore, spindle abnormalities were not the consequence of reduced cyclin B2 levels as coexpressing 9A-Hec1 in Hec1-depleted oocytes completely restored cyclin B2 levels (Figure S4B). Thus, specifically impairing Hec1’s kinetochore function compromised late-stage spindle assembly, leaving earlier spindle stages largely intact.

We reasoned that if Hec1 was mediating its effects on late-stage spindle assembly via its kinetochore-related properties then an independent approach for disrupting kinetochore function might also be expected to produce a similar pattern of spindle assembly defect. We therefore examined the effect of depleting the kinetochore motor protein, CENP-E, which we previously showed localizes to kinetochores in mouse oocytes (Gui and Homer, 2012). We used a *mCENP-E*-targeting morpholino (CENPEMO) that we recently characterized (Gui and Homer, 2012) (Figure S4A), which, unlike Hec1 depletion, did not reduce cyclin B2 levels; indeed, there was a trend toward cyclin B2 stabilization after CENP-E depletion (Figure S4B). Overall, we found that the majority of CENP-E-depleted oocytes were able to form bipolar spindles by 8 hr post-GVBD (Figures 6C and S4C), consistent with our previous findings (Gui and Homer, 2012). Notably, however, using the criteria we applied here, we found that CENP-E depletion led to detectable abnormalities in spindle assembly (Figures 6A–6C). Significantly, as with coexpression of 9A-Hec1 in Hec1-depleted oocytes, although a small proportion (∼3%) of CENP-E-depleted oocytes exhibited sustained defects in early-stage spindle assembly (Figures 6C and S4D), defects in the latter stages were about 3-fold higher than in wild-type oocytes, affecting almost 20% of oocytes (Figures 6C and S4E). Thus, findings in CENP-E-depleted oocytes independently corroborate our 9A-Hec1 data in showing that kinetochores are especially important during the latter stages of acentrosomal spindle assembly.

Taken together therefore, whereas Hec1-dependent cyclin B2 stabilization contributes primarily to the initial stages of spindle assembly required for individualization, Hec1’s kinetochore function is especially important for completing the latter stages of bipolarization.

### Hec1 Stabilizes Cyclin B2 against APC^Cdh1^-Mediated Proteolysis before Becoming Uncoupled from Cyclin B2 in Late MI

We asked whether Hec1’s requirement for cyclin B2 stability might be a reflection of a more general role for kinetochores in this process. Strikingly, however, we observed that at the GV stage, the bulk of Hec1 did not colocalize with chromosomes or with inner kinetochore proteins. Instead, Hec1 mostly colocalized with cyclin B2 external to the GV (Figure S5A). It was only after GVBD that Hec1 began to clearly colocalize with inner kinetochore proteins as punctate foci, thereafter retaining this pattern of localization throughout M phase (Figure S5B). Thus, at the GV stage, the majority of Hec1 localizes external to the GV with cyclin B2 and not at sites of kinetochore assembly. Furthermore, given that Hec1 is indispensable to outer kinetochore plate integrity (DeLuca et al., 2005), we can also infer that in mouse oocytes, kinetochore assembly is incomplete in prophase. An important implication of these findings is that the effect of Hec1 on cyclin B2 is unlikely to reflect an intrinsic kinetochore-mediated function.

In order to further explore a potential role for kinetochores in stabilizing cyclin B2, we examined CENP-E-depleted oocytes. In stark contrast to Hec1-depleted oocytes, CENP-E depletion did not compromise GVBD; indeed, there was a small increase in spontaneous GVBD during culture in IBMX-treated medium after CENP-E depletion (3.2% ± 1.5% [n = 48] versus <1% in wild-type) that could be related to the trend we observed previously toward increased cyclin B2 levels (Figure S4B). Given that Hec1 depletion and CENP-E depletion have contrasting effects on cyclin B2 and on GVBD, we conclude that in oocytes, promoting the G2-M transition via cyclin B2 is not a universal function of kinetochores.

**Figure 7:**
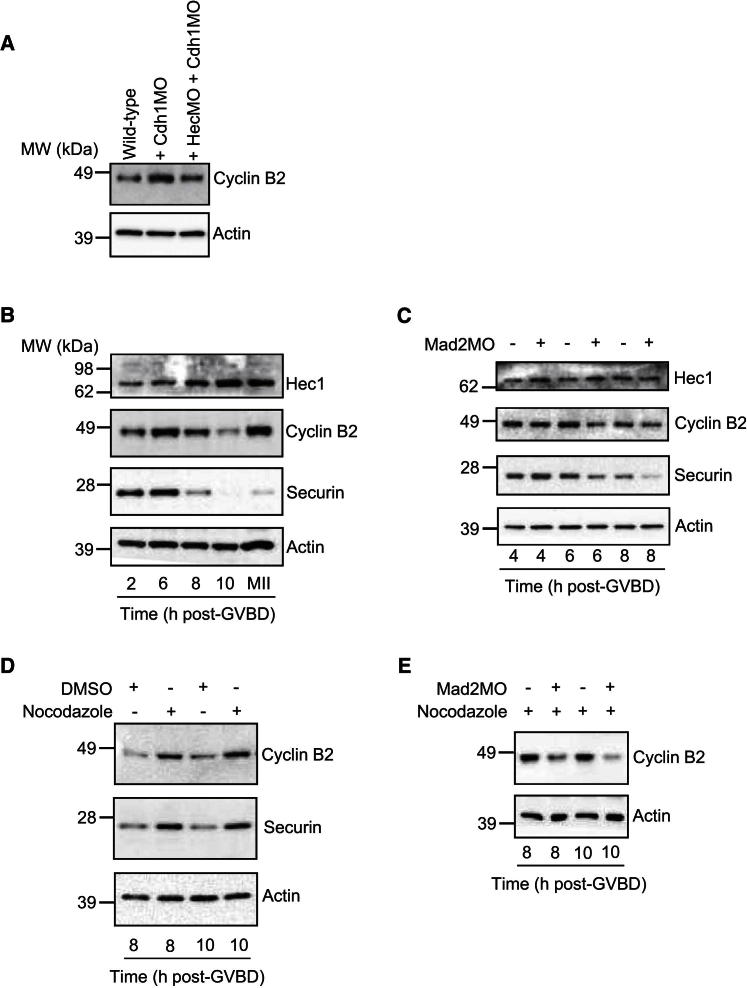
Hec1 Stabilizes Cyclin B2 against APC^Cdh1^ in Early MI, whereas Cyclin B2 Is Degraded by APC^Cdc20^ in Late MI and Hec1 Remains Stable (A) Immunoblot of cyclin B2 in wild-type, Cdh1-depleted (+ Cdh1MO), and Hec1- and Cdh1-double-depleted (+ HecMO + Cdh1MO) oocytes. (B) Immunoblot of Hec1, cyclin B2, and securin during MI and after becoming arrested at metaphase of meiosis II (MII) in wild-type oocytes. (C) Immunoblot of Hec1, cyclin B2, and securin in wild-type oocytes and oocytes depleted of Mad2 using a well-characterized *mMAD2*-targeting morpholino (Mad2MO) (Gui and Homer, 2012; Homer et al., 2005a, 2005b, 2009). Note that cyclin B2 and securin decline by 8 hr post-GVBD in controls, whereas this decline occurs 2 hr earlier following Mad2 depletion. (D) Wild-type oocytes were transferred to culture medium containing either DMSO or nocodazole at 4 hr post-GVBD following which samples were collected at 8 and 10 hr post-GVBD and immunoblotted for cyclin B2 and securin. Note that cyclin B2 and securin levels are both higher following nocodazole treatment. (E) Mad2-depleted and wild-type oocytes were transferred to nocodazole-treated medium at 4 hr post-GVBD after which samples were collected at 8 and 10 hr post-GVBD and immunoblotted for cyclin B2. Note the reduced cyclin B2 levels in Mad2-depleted oocytes, indicating that the SAC is required for cyclin B2 stabilization following spindle depolymerization. Samples contained 50 oocytes, and actin served as a loading control. Data are representative of at least two experimental replicates. See also Figure S5.

During prophase and early prometaphase, APC^Cdh1^ is the active APC species in oocytes, whereas by late prometaphase, APC^Cdc20^ predominates (Homer et al., 2009; Homer, 2011; Reis et al., 2006, 2007). The foregoing data showed that Hec1 was required for stabilizing cyclin B2 during G2 and early prometaphase, coincident therefore with the phase in which APC^Cdh1^ is active. This suggested that APC^Cdh1^ could provide an explanation for Hec1’s influence on cyclin B2 stability, for instance, by guarding against APC^Cdh1^-mediated cyclin B2 proteolysis. Consistent with cyclin B2 being a APC^Cdh1^ substrate in oocytes, depletion of Cdh1 stabilized cyclin B2 (Figure 7A) and an exogenous ^Δ*D-box*^*cyclin B2* cRNA bearing a mutation in cyclin B2’s APC destruction motif known as the D-box (Chapman and Wolgemuth, 1993) was more stable than exogenous wild-type cyclin B2 in GV-stage oocytes (Figure S5C). In order to examine whether reduced levels of Hec1 exposes cyclin B2 to APC^Cdh1^-mediated proteolysis, we asked whether reducing APC^Cdh1^ activity in Hec1-depleted oocytes might be able to stabilize cyclin B2. Significantly, we found that codepletion of Cdh1 in Hec1-depleted oocytes was indeed able to restore cyclin B2 to levels above that found in wild-type oocytes (Figure 7A). Importantly, therefore, when Hec1 is lacking, cyclin B2 becomes vulnerable to APC^Cdh1^-mediated destruction.

Given that Hec1 was recently shown to be an APC^Cdh1^ substrate (Li et al., 2011), we asked whether Hec1 could be stabilizing cyclin B2 by acting as a competitive substrate inhibitor. Securin and cyclin B1, two other APC^Cdh1^ substrates in GV-stage oocytes, have been shown to involved in this mode of regulation; securin is important for cyclin B1 stability by competing with cyclin B1 as a substrate for APC^Cdh1^, in effect acting as a cyclin B1-specific APC^Cdh1^ inhibitor (Marangos and Carroll, 2008). A characteristic feature of this mode of regulation is that increasing or decreasing securin leads to parallel changes in cyclin B1 (Marangos and Carroll, 2008). Significantly, however, although Hec1 depletion was accompanied by cyclin B2 reduction, increasing Hec1 did not induce detectable increases in cyclin B2 (Figure S5D; see also Figure S1C), consistent with which, Hec1 overexpression did not augment GVBD (data not shown). Significantly, however, we found that Hec1 coimmunoprecipitated cyclin B2, but not cyclin B1 (Figures S5E and S5F). Thus, our data do not support substrate competition as the underlying mechanism by which Hec1 stabilizes cyclin B2 against APC^Cdh1^. Instead, our finding that Hec1 binds to cyclin B2 raises the possibility that through complex formation, Hec1 might make cyclin B2 less accessible to APC^Cdh1^. Notably, the lack of observed binding between Hec1 and cyclin B1 would be consistent with our observation that Hec1 does not influence cyclin B1 stability.

In stark contrast to early MI when cyclin B2 levels increased, by late MI when APC^Cdc20^ is active, we found that cyclin B2 declined precipitously (Figure 7B). The SAC modulates APC^Cdc20^ activity in late MI and so determines the timing of APC^Cdc20^-mediated securin and cyclin B1 destruction (Homer et al., 2005b; McGuinness et al., 2009; Niault et al., 2007; Reis et al., 2007). Notably, cyclin B2 decline coincided with the timing of securin destruction pointing to APC^Cdc20^-mediated cyclin B2 destruction. Consistent with this, Mad2 depletion (which induces precocious APC^Cdc20^ activation) (Homer et al., 2005b) accelerated the onset of cyclin B2 destruction in parallel with securin (Figure 7C). In contrast, nocodazole-induced spindle depolymerization (which inhibits APC^Cdc20^ through SAC activation) (Homer et al., 2005a) stabilized cyclin B2 in a Mad2-dependent manner (Figures 7D and 7E). Highly significantly, cyclin B2 destruction occurred while Hec1 remained stable (Figure 7B). Intriguingly, therefore, Hec1 is required for stabilizing cyclin B2 against APC^Cdh1^ during prophase and early prometaphase and gives way to APC^Cdc20^-mediated cyclin B2 destruction in late MI, the timing of which is set by the SAC.

## Discussion

Our results show that modest reductions in cyclin B2 accompanying Hec1 depletion substantially impair MI, contrasting sharply with the minimal effects observed in mitosis following more severe depletions (Bellanger et al., 2007; Gong et al., 2007). Our findings further indicate that this arises because the oocyte’s environment limits the ability of other Cdk1-activating cyclins, such as cyclin B1, to adequately cover for cyclin B2 loss as occurs in mitosis (Bellanger et al., 2007). Significantly, a major feature of mouse oocytes not shared with mitosis is APC^Cdh1^-mediated cyclin B1 proteolysis during prophase (Reis et al., 2006). It is possible that within such an environment of cyclin B1 restraint, increased dependency is placed on cyclin B2 to prevent basal Cdk1 tone from dropping too low and to reinforce cyclin B1 pathways during Cdk1 activation. Analogously, APC^Cdh1^-directed Cdc20 proteolysis during prometaphase (Reis et al., 2007) likely explains why meiosis is impaired in oocytes from mice with reduced Cdc20 expression, whereas mitosis remains unperturbed (Jin et al., 2010). We note, however, that a minority of Hec1-depleted oocytes do enter MI, albeit with severely delayed kinetics. Thus, although of low efficiency, compensatory mechanisms nevertheless emerge in oocytes, perhaps explaining why *CCNB2*-knockout animals exhibit reduced fertility but are not sterile (Brandeis et al., 1998). Indeed, compared with fully grown oocytes acutely depleted of cyclin B2 by morpholinos (these data), the defect in *CCNB2*-knockout oocytes may be less severe as compensatory mechanisms have greater opportunity to emerge during their 2- to 3-week-long growth phase. These issues highlight the importance of further ascertaining the physiological relevance of this mode of regulation by examining oocytes subjected to more chronic reductions in Hec1, for instance, when Hec1 is specifically ablated from the start of the oocyte’s growth phase (see McGuinness et al., 2009).

Surprisingly, our data reveal that cyclin B2 is regulated by Hec1 specifically during prophase and early prometaphase, thereby uncovering a role for Hec1 at the G2-M transition and in early-stage acentrosomal spindle assembly. We find that Hec1 is required to stabilize cyclin B2 against APC^Cdh1^. However, it is currently unclear exactly how Hec1 affords cyclin B2 this protection. Although Hec1 is also an APC^Cdh1^ substrate (Li et al., 2011), our data do not support competitive substrate inhibition—by which securin stabilizes cyclin B1 against APC^Cdh1^ in GV-stage oocytes (Marangos and Carroll, 2008)—as the mechanism by which Hec1 stabilizes cyclin B2. Hec1 overexpression has previously been found to stabilize cyclin by inhibiting MSS1, a proteasomal component downstream of the APC (Chen et al., 1997). Although this provides one potential means by which Hec1 could stabilize cyclin B2 in oocytes, it neither readily explains the lack of similar effect on cyclin B1 nor does such a model conform with our finding that cyclin B2 levels are not increased by Hec1 overexpression. As expected for critically important regulators like cyclins, multiple inputs (of which proteolysis is but one) are certain to be employed for setting steady-state cyclin B2 levels. Indeed, in contrast to the positive effect of Hec1 on cyclin B2 levels found here, *CCNB2* expression has also been shown to be subject to stringent negative regulation at the transcriptional level by the tumor suppressor, Menin (Wu et al., 2010). Competing inputs could explain why the loss of Hec1 exposes cyclin B2 to proteolysis on the one hand and why, on the other hand, Hec1 overexpression does not induce a linear increase in cyclin B2.

In contrast with mitosis, in which Hec1 and Nuf2 move through the nuclear membrane to become localized to kinetochores in G2 prior to NEBD (Hori et al., 2003), we find that Hec1 does not colocalize with kinetochores at the GV stage but instead localizes external to the GV. Given that the GV is the focus of maximal APC^Cdh1^ activity in mouse oocytes (Holt et al., 2010), an appealing model is that Hec1 stabilizes cyclin B2 by virtue of spatially sequestering it away from APC^Cdh1^ through direct binding. Our findings are entirely consistent with data from budding and fission yeast showing that Ndc80 is undetectable at kinetochores during late meiotic prophase (Asakawa et al., 2005; Miller et al., 2012). In budding yeast, close temporal coordination between the kinetochore assembly and Cdk activation pathways is important for setting up the MI-specific pattern of reductional chromosome segregation (Miller et al., 2012). Our findings point to another dimension of coordinated interplay between kinetochore proteins and Cdk in meiosis, this time important for the G2-M transition.

As MI progresses, the influence of Hec1 on cyclin B2 stability wanes—even though Hec1 remains stable, cyclin B2 becomes subject to marked APC^Cdc20^-directed destruction in late MI—and Hec1’s conventional kinetochore-based role becomes more apparent. Thus, after bivalents have individualized following passage through the early stages of spindle assembly, Hec1 appears important for kinetochore reorientation as 9A-Hec1 expression results in multiple compact bivalents. Strikingly, when reorientation is impaired, either with 9A-Hec1 or after CENP-E depletion, it is the latter stages of spindle bipolarization that are predominantly affected. Significantly therefore, in oocytes in which spindle bipolarity is not predefined by a centrosomal pair, our data suggest that kinetochores oriented to face in opposite directions promote later stages of spindle bipolarization reminiscent of the impaired bipolarization observed in mitotic cells lacking centrosomes in which multiple juxtaposed kinetochores have been induced (Loncarek et al., 2007). It may be that equatorially located linear-shaped bivalents, which would be facilitated by reorientation, reinforce central spindle robustness, recently shown to be a crucial determinant of bipolarization in mouse oocytes (Breuer et al., 2010). Our findings do not in any way rule out other roles for Hec1 in acentrosomal spindle assembly, for instance, through Hice1 (Wu et al., 2009) or by stabilizing other APC^Cdh1^ substrates, such as TPX2, depletion of which induces a phenotype reminiscent of that observed after Hec1 depletion (Brunet et al., 2008). Overall therefore, by extending Hec1’s functions beyond M phase, these data identify Hec1 as a pivotal node for integrating M phase entry with proper M phase progression.

## Experimental Procedures

### Oocyte Collection, Culture, and Drug Treatment

Oocytes were isolated from 4- to 6-week-old MF1 mice and cultured as previously described (Gui and Homer, 2012; Homer et al., 2005b, 2009) (see the Supplemental Experimental Procedures).

### Microinjection of Morpholinos and cRNAs

GV-stage oocytes were microinjected with morpholinos and maintained in medium supplemented with IBMX for 24 hr before being washed into IBMX-free medium to induce GVBD (see the Supplemental Experimental Procedures).

For making cRNAs, cDNAs encoding hHec1, 9A-Hec1-GFP (a kind gift from Dr. J. De Luca, Colorado State University, Fort Collins, CO, USA), cyclin B2, and cyclin B2-GFP were used to generate linearized templates for in vitro transcription using the mMESSAGE mMACHINE kit (Ambion, Austin, TX, USA) (see the Supplemental Experimental Procedures). Following microinjection of cRNA, GV-stage oocytes were maintained for a minimum of 2 hr in IBMX-treated medium to facilitate translation.

### Western Blotting

Antibodies against securin, actin, cyclin B1, Cdh1, and CENP-E were described previously (Gui and Homer, 2012; Homer et al., 2009; Marangos and Carroll, 2008; Reis et al., 2007). For detecting Hec1, we used panHec1, a rabbit polyclonal antibody raised against mHec1 (Diaz-Rodríguez et al., 2008; a kind gift from Dr. R. Benezra, Memorial Sloan Kettering Cancer Center, New York) (see the Supplemental Experimental Procedures).

### Histone H1 Kinase Assays

Kinase assays were performed using groups of 15 oocytes based on a previously described method (Kubiak et al., 1993). Proteins were resolved on 4%–12% Bis-Tris gels (NuPAGE; Invitrogen, Carlsbad, CA, USA), after which the incorporation of [^32^P] was analyzed using a PhosphorImager (GE Healthcare, Amersham Place, Buckinghamshire, UK) (see the Supplemental Experimental Procedures).

### Immunocytochemistry

Primary antibodies included β-tubulin (Sigma-Aldrich, St. Louis), ACA (ImmunoVision, Springdale, AR, USA) (Duncan et al., 2009), and panHec1. DNA was stained using Hoechst 33342 (10 μg/ml; Sigma-Aldrich). Images were captured using a LSM 510 META confocal microscope, processed using MetaMorph software, and assembled into panels using Adobe Photoshop (see the Supplemental Experimental Procedures).

## Supplemental Information

Document S1. Figures S1–S5 and Supplemental Experimental Procedures
