# Supplementary material for: Hec1-Dependent Cyclin B2 Stabilization Regulates the G2-M Transition and Early Prometaphase in Mouse Oocytes
Source: Dev Cell. 2013 Apr 15;25(1):43–54. doi: 10.1016/j.devcel.2013.02.008 (PMC3659288; doi:10.1016/j.devcel.2013.02.008)
Supplement: Document S1. Figures S1–S5 and Supplemental Experimental Procedures [file mmc1.pdf]

Developmental Cell, Volume *25*

Supplemental Information

Hec1-Dependent Cyclin B2 Stabilization Regulates the G2-M Transition and Early Prometaphase in Mouse Oocytes

Liming Gui and Hayden Homer

Inventory of Supplemental Information

We provide 5 supplemental figures:

Figure S1 is related to Figure 2 and shows quantification of cyclin B2 reduction after Hec1 depletion and the stabilising effect of co-expressed hHec1 on cyclin B2 in Hec1 depleted oocytes.

Figure S2 is related to Figure 3 and shows a comparison of cyclin B1-GFP and cyclin B2-GFP expression levels under differing experimental conditions.

Figure S3 is related to Figure 4 and shows differences in K-fibre content in wild-type versus Hec1 depleted oocytes; different phases of kinetochore re-orientation and spindle assembly in wild-type oocytes, and; normal bipolar spindle structure in mock depleted oocytes and Hec1 depleted oocytes rescued with hHec1.

Figure S4 is related to Figure 6 and shows morpholino-induced CENP-E depletion, a lack of cyclin B2 decline either when 9A-Hec1 is co-expressed in Hec1 depleted oocytes or after CENP-E depletion and various spindle morphologies observed after CENP-E depletion.

Figure S5 is related to Figure 7 and shows Hec1 co-localisation first with cyclin B2 external to the GV and then with kinetochores after GVBD; relative stabilisation of cyclin B2 after D-box mutation but not after Hec1 over-expression, and; Hec1 binding to cyclin B2.

Supplemental Experimental Procedures

Supplemental References

Supplemental Information

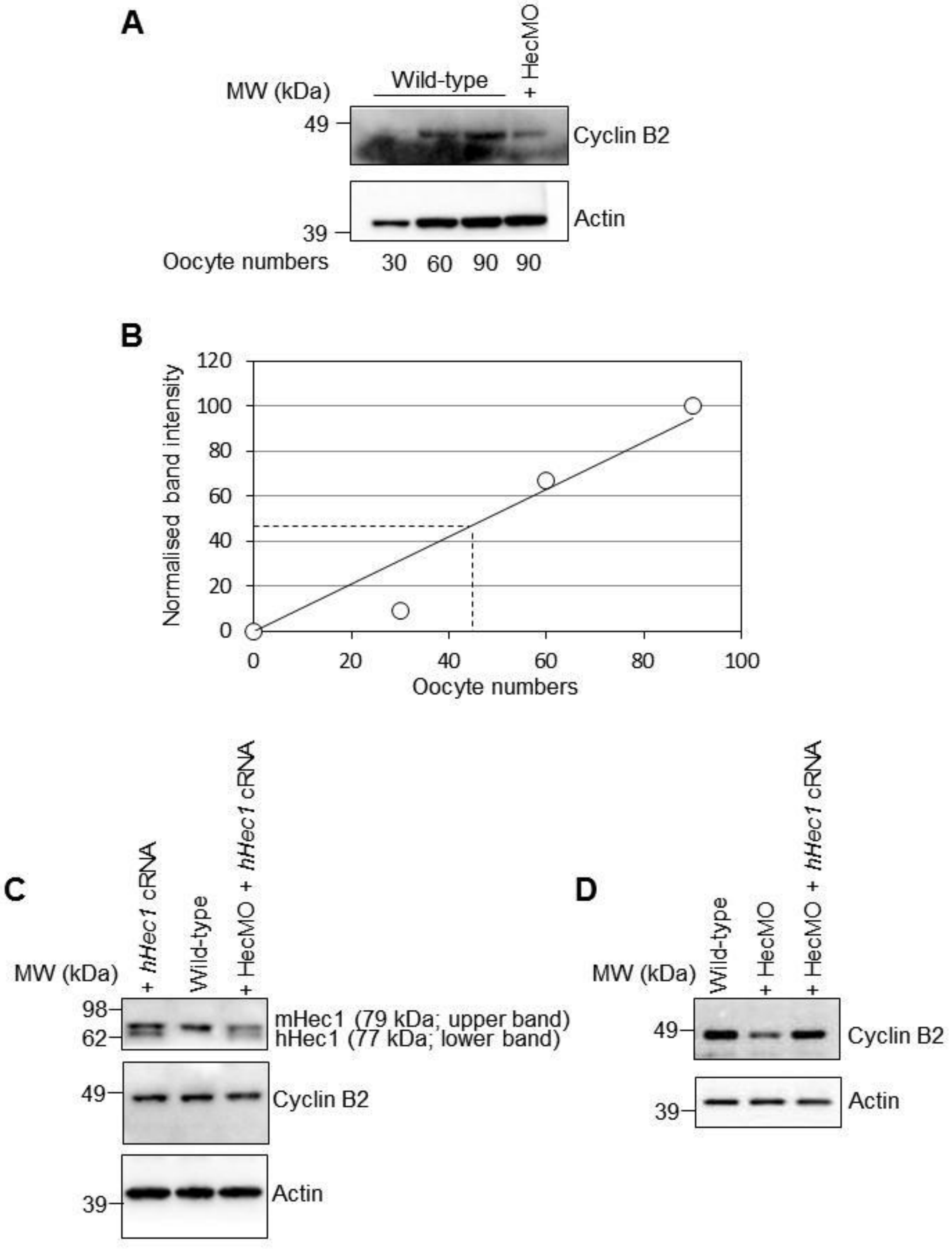

Supplementary Figure S1_Gui & Homer, Related to Figure 2

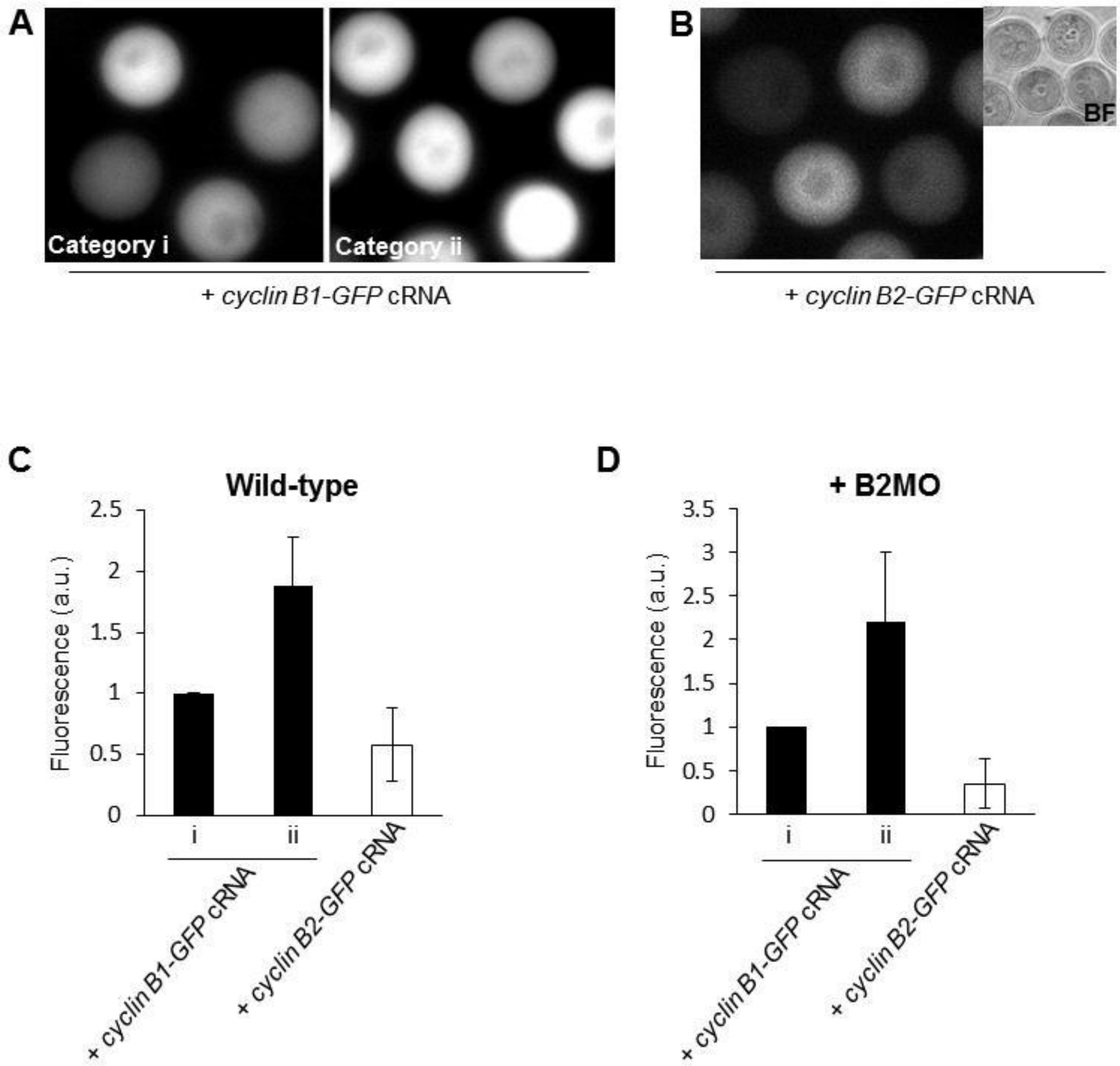

Supplementary Figure S2_Gui & Homer, Related to Figure 3

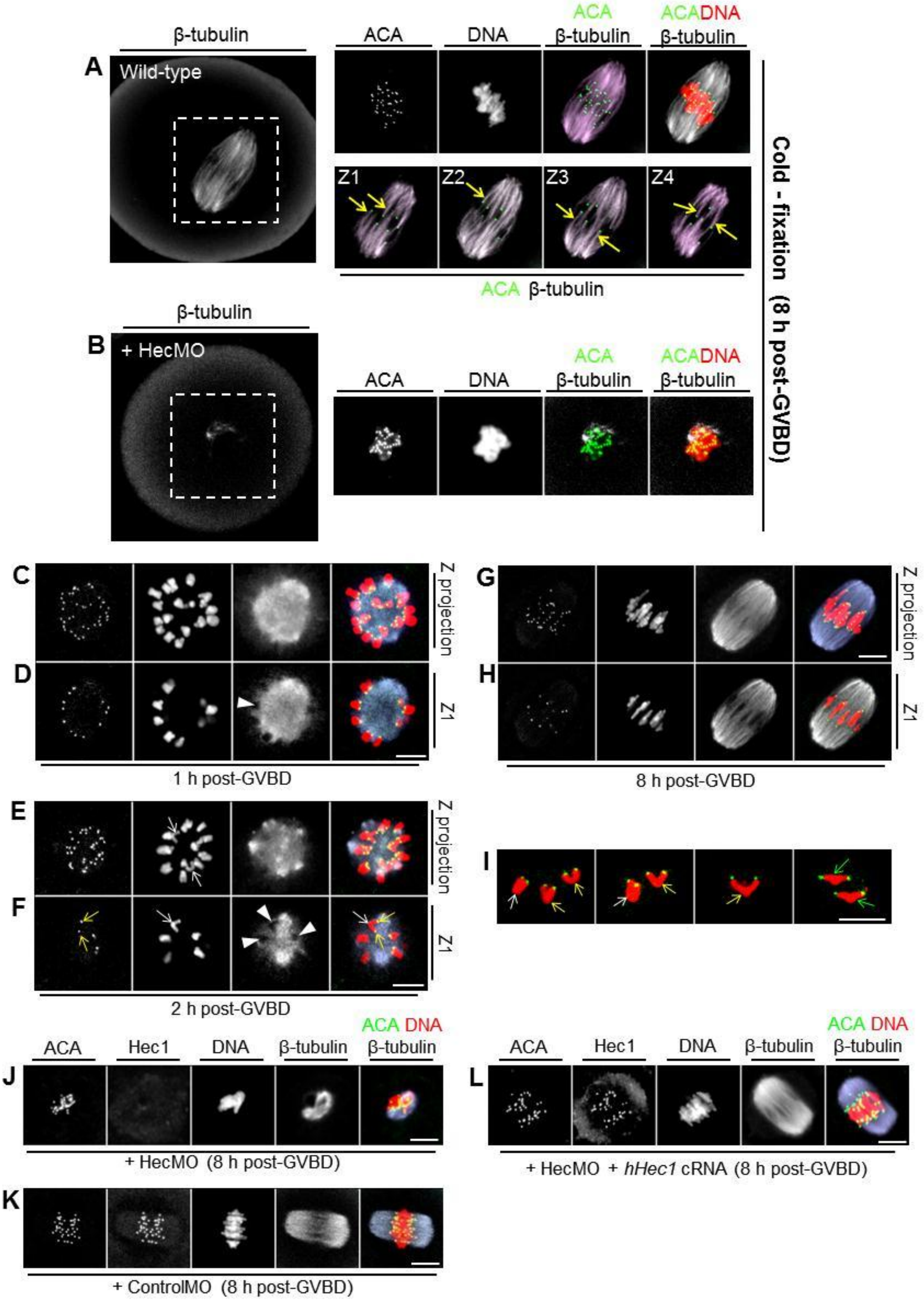

Supplementary Figure S3_Gui & Homer, Related to Figure 4

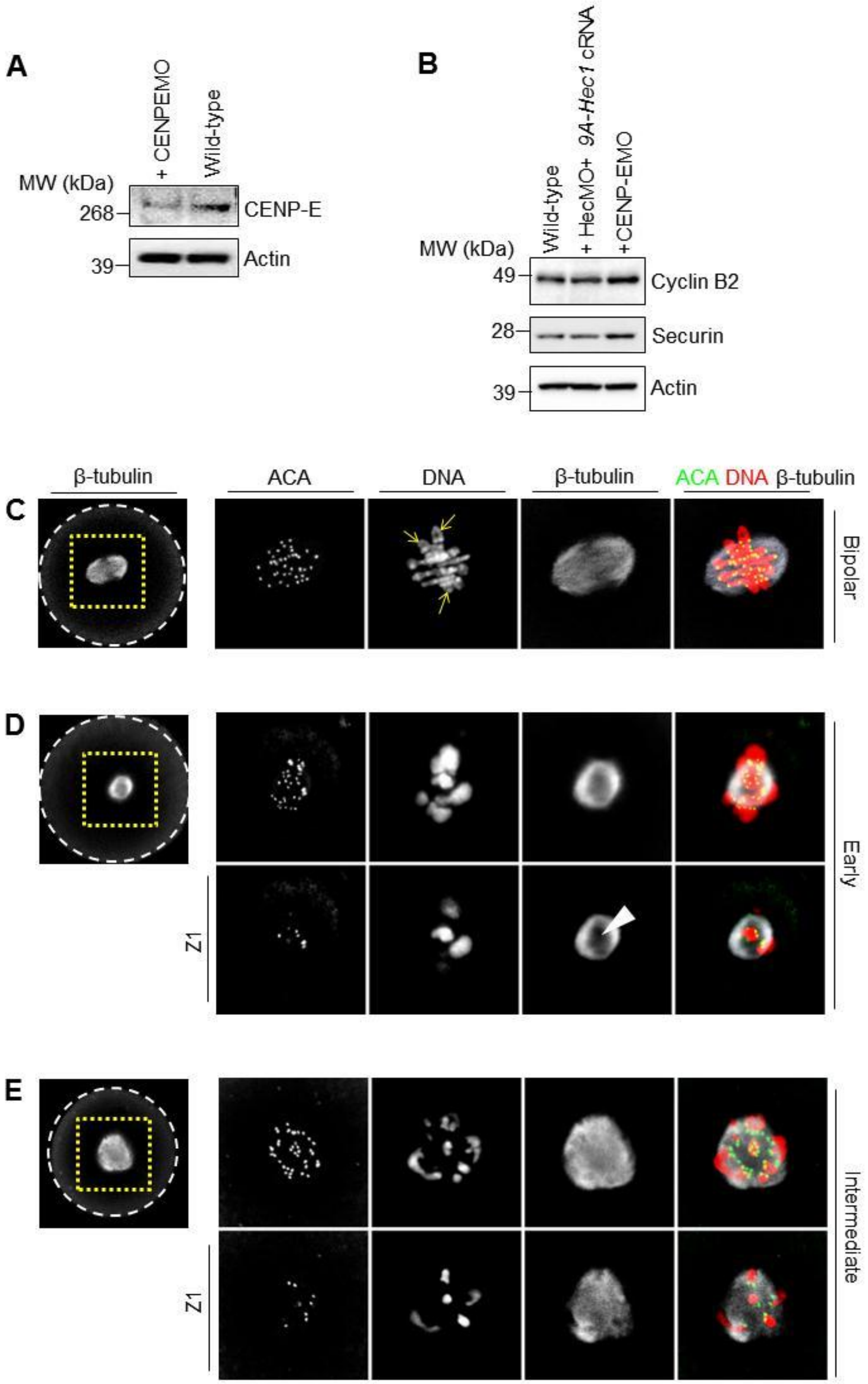

Supplementary Figure S4_Gui & Homer, Related to Figure 6

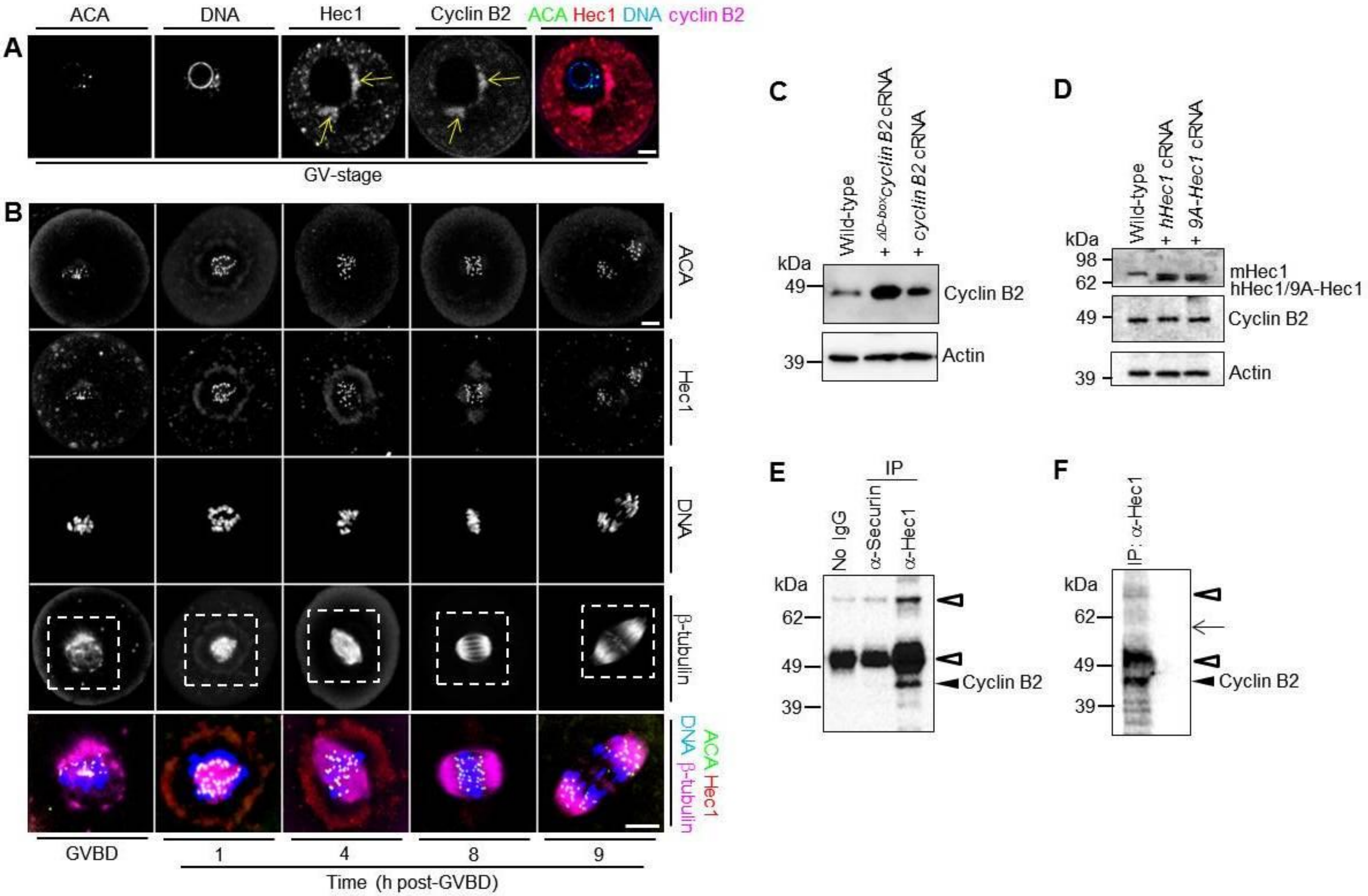

Supplementary Figure S5_Gui & Homer, Related to Figure 7

Supplemental Figure Legends

Figure S1 Impact of Hec1 depletion and expression of Hec1 from exogenous *hHec1* cRNA on cyclin B2 levels, Related to Figure 2.

(A) Immunoblot of cyclin B2 in 30, 60 and 90 wild-type GV-stage oocytes alongside 90 GV-stage oocytes microinjected with HecMO. Note that the cyclin B2 signal intensity in HecMO-injected oocytes falls between that produced by 30 and 60 wild-type oocytes.

(B) Quantifying cyclin B2 levels after Hec1 depletion. Cyclin B2 band intensities for control oocytes were quantified by densitometric analysis as we did previously (Homer et al., 2009), normalised using 90 oocytes as 100 % and plotted against oocyte numbers to generate a calibration curve. The dotted line indicates where the band intensity of the HecMO-injected oocytes intersected the calibration curve.

(C) GV-stage oocytes were microinjected with either *hHec1* cRNA or a combination of HecMO and *hHec1* cRNA and maintained for 24 h in 50 µM IBMX before being blotted alongside wild-type GV-stage oocytes for Hec1 and cyclin B2. Note that mHec1 migrates slower than hHec1 as described previously (Diaz-Rodríguez et al., 2008).

(D) In order to more clearly illustrate the stabilising effect of hHec1 on cyclin B2 levels after Hec1 depletion, shown is an immunoblot of wild-type oocytes, Hec1 depleted oocytes (+ HecMO) and Hec1 depleted oocytes co-expressing hHec1 (+ HecMO + *hHec1* cRNA).

Figure S2 Using GFP-tagged cyclin B1 and cyclin B2 constructs to estimate levels of protein expression, Related to Figure 3.

The cyclin B1-GFP and cyclin B2-GFP constructs enabled us to estimate the levels of cyclin B1 and cyclin B2 over-expression based on oocyte fluorescence as we did previously (Homer et al., 2005b).

(A-C) Wild-type GV-stage oocytes were microinjected either with (a) *Cyclin B1-GFP* cRNA

and maintained in medium treated with high concentrations of IBMX (200 µM) for either 2 h (Category i; n = 26) or 6 h (Category ii; n = 18) or with; (b) *Cyclin B2-GFP* cRNA (n = 26) and maintained in IBMX (200 µM) for 2 h. High IBMX concentrations were used to allow time for either cyclin B1-GFP or cyclin B2-GFP translation whilst at the same time maintaining G2-prophase arrest. Total oocyte GFP fluorescence was then measured and normalized to the mean fluorescence value for Category i. Note that 6 h of cyclin B1-GFP translation produces about double the fluorescence (and hence roughly double the concentration of cyclin B1-GFP) of that produced after 2 h of translation ($P = 0.038$). Significantly, 2 h of protein translation produced somewhat lower levels of cyclin B2-GFP than cyclin B1-GFP (B and C). Inset in (B) shows the corresponding brightfield (BF) image of the cyclin B2-GFP-expressing oocytes.

(D) The above experiment was repeated in oocytes in which cyclin B2 was depleted using B2MO. Based on GFP fluorescence intensities, we found very similar relative expression levels of cyclin B1-GFP (after allowing for either 2 h [n = 22] or 6 h [n = 25] of expression in IBMX) and cyclin B2-GFP (after 2 h of expression; n = 36) on a cyclin B2 knockdown background as observed in wild-type oocytes (compare with C) albeit there was a small trend towards lower absolute cyclin B2-GFP expression after cyclin B2 depletion. Significantly however, even with such comparatively low levels of cyclin B2-GFP expression, GVBD could readily be restored in cyclin B2 depleted oocytes whereas considerably higher levels of cyclin B1-GFP expression were required to produce a similar effect (see Figure 3B).

Figure S3 Features of chromosomes and spindles in wild-type, Hec1 depleted and mock depleted oocytes and in Hec1 depleted oocytes co-expressing hHec1, Related to Figure 4.

(A and B) Hec1 depleted oocytes are deficient in cold-stable K-fibres. Representative confocal images of immunostained wild-type (A) and Hec1 depleted (B) oocytes fixed after

transient exposure to ice-cold conditions for depolymerising non K-fibre microtubules. Whole oocyte Z projection images in the β-tubulin channel are shown on the left whilst panels to the right depict Z sections (Z1-Z4) though the region demarcated by the dashed white boxes. After cold-fixation, kinetochore-attached microtubule bundles become more prominent in wild-type oocytes (yellow arrows, A) whereas in Hec1 depleted oocytes, the bulk of microtubules become depolymerised (B) consistent with Hec1's known role in K-fibre formation (DeLuca et al., 2005).

(C-I) Following individualisation bivalents undergo "stretching" and kinetochore re-orientation as the spindle bipolarises. (C-F) Representative confocal Z projections (C and E and G) and single confocal Z sections referred to as Z1 (D and F and H) of oocytes immunostained for kinetochores (ACA), DNA and microtubules (β-tubulin) at the stages shown during MI. (C and D) Post-individualisation, bivalents become discernible as individual chromosomes and are distributed on the surface of the microtubule ball. Note that on single confocal sections (D) it is clear that all bivalents possess closely juxtaposed kinetochores and occupy peripheral microtubule pockets (arrowhead). (E and F) By 2 h post-GVBD, some bivalents relocate towards the spindle interior where, on single Z-sections (F), they can be seen to be completely surrounded within microtubule "windows" (arrowheads). Note that these windows surround individual bivalents whereas spindle windows occurring in Hec1 depleted oocytes are much larger and enclose clusters of multiple chromosomes (see Figure 4E). Importantly, a few bivalents begin to take on a V-shape (white arrow) with kinetochores located at the extremities of each arm distal to the apex of the V (yellow arrows). Thus, whilst the spindle is still predominantly spherical, kinetochores are starting to re-orient. (G and H) By 8 h post-GVBD, almost all bivalents are extended with kinetochores directed towards opposite spindle poles. (I) Progressive stages of bivalent stretching and kinetochore re-orientation are illustrated by immunostaining. Compact bivalents (white

arrows) convert via a V-shaped intermediate (yellow arrows) into an extended configuration (green arrows).

(J-L) Shown are representative confocal Z projection images of a Hec1 depleted oocyte (J), a mock depleted oocyte (K) and a Hec1 depleted oocyte co-expressing hHec1 (L) immunostained for kinetochores (ACA), Hec1, DNA and microtubules (β-tubulin) at 8 h post-GVBD.

Scale bars, 10 µm.

Figure S4 Effect of co-expressing 9A-Hec1 on cyclin B2 expression in Hec1 depleted oocytes and effect of CENP-E depletion on cyclin B2 and spindle assembly, Related to Figure 6.

(A) GV-stage oocytes were microinjected with CENPEMO and maintained in IBMX for 24 h before being blotted alongside wild-type oocytes for CENP-E as described previously (Gui and Homer, 2012). 200 oocytes per sample.

(B) Immunoblot of cyclin B2 and securin in Hec1 depleted oocytes co-expressing 9A-Hec1 from exogenous cRNA, CENP-E depleted oocytes and wild-type oocytes at the GV-stage. 50 oocytes per sample. Note that after CENP-E depletion there was a trend towards increased cyclin B2 levels. This would be consistent with reduced APC^{Cdh1} activity as CENP-E has previously been shown to be important for BubR1 stability in oocytes (Gui and Homer, 2012), which in turn is required for sustaining Cdh1 levels (Homer et al., 2009). In keeping with this, we also observed an increase in securin after CENP-E depletion akin to that observed as a result of the reduced APC^{Cdh1} activity that accompanies BubR1 depletion (Homer et al., 2009).

(C-E) Confocal images of CENP-E depleted oocytes immunostained for kinetochores (ACA), Hec1, DNA and microtubules (β-tubulin) at 8 h post-GVBD showing the different

morphologies observed (determined using criteria set out in Figure 5) including normal bipolar (C), early stage (D) and intermediate (E) spindle morphologies. Note that although the majority of bivalents were extended in CENP-E depleted oocytes possessing bipolar spindles, a prominent feature in some cases was bivalents with a compact configuration (yellow arrows, C) consistent with previous findings (Gui and Homer, 2012). In oocytes with an early stage morphology, chromosomes form clumps (D) and individual Z sections reveal spindle windows (white arrowhead, Z1, D). When an intermediate morphology is present (E) bivalents have individualised but most are of a compact configuration and spindle windows are not a prominent feature on individual Z sections. Panels to the left are whole-oocyte images in the β-tubulin channel with the dashed white circles outlining the oocyte whilst panels to the right are magnified images of the region enclosed by the dashed yellow squares.

Figure S5 Relating Hec1 dependent cyclin B2 stabilisation to sub-cellular localisation and protein-protein interaction, Related to Figure 7.

(A and B) Shown are representative confocal Z projection images of wild-type oocytes immunostained for kinetochores (ACA), Hec1, DNA and either cyclin B2 (A) or microtubules (β-tubulin)(B) at the GV-stage (A) and at the stages shown post-GVBD (B). Note that at the GV-stage, Hec1 and cyclin B2 localise external to the GV and exhibit concentrated zones of co-localisation (yellow arrows, A). Significantly, there is no clear co-localisation between ACA and Hec1 until immediately after GVBD, a pattern that then persists through to anaphase I. Scale bars, 10 µm.

(C) Immunoblot of cyclin B2 in wild-type oocytes and in oocytes co-injected with cRNA encoding either a D-box mutated cyclin B2 ($^{\Delta D\text{-}box}$*cyclin B2* cRNA) or wild-type cyclin B2. 50 oocytes per sample.

(D) Immunoblot of cyclin B2 and Hec1 in wild-type oocytes and in oocytes co-injected with cRNA encoding either wild-type hHec1 or 9A-hHec1. As before (see Figure S1C), mHec1 migrates more slowly than hHec1. Note that cyclin B2 levels do not change with over-expression of either hHec1 or 9A-Hec1 (see also Figure S1C).

(E) Immunoblot of cyclin B2 in Hec1 and securin immunoprecipitates. 200 oocytes per sample. Note that cyclin B2 (black arrowhead) co-precipitated with Hec1 but not with securin. As a negative control, an oocyte lysate (200 oocytes) was incubated with beads alone (lane 1, no IgG). Two bands observed in Hec1 and securin immunoprecipitates also appeared in this lane (open arrowheads) indicating that they represent non-specific protein binding with beads.

(F) Immunoblot of cyclin B1 and cyclin B2 in Hec1 immunoprecipitates. The membrane was first probed with anti-cyclin B1 before being probed with anti-cyclin B2. Note that no band is seen in cyclin B1's predicted position (55-60 kDa, arrow) whereas a clear band is seen at cyclin B2's predicted position (~45 kDa, black arrowhead) as seen before in (E). As in (E), two non-specific bands (open arrowheads) are again observed.

Supplemental Experimental Procedures

Oocyte collection, culture and drug treatment

Ovaries were isolated from 4-6 week-old female mice of the MF1 strain 46-48 h after intra-peritoneal injection of 7.5-10 international units (IU) of pregnant mare's serum gonadotrophin (PMSG; Intervet). Ovaries were placed in a Petri dish with pre-warmed (37°C) M2 medium (Sigma, Poole, UK) supplemented with 50 µM 3-isobutyl-1-methylxanthine (IBMX; Sigma) so as to prevent oocytes from undergoing germinal vesicle breakdown (GVBD). Fully grown, GV-intact oocytes were released into IBMX-treated M2 medium by puncturing antral follicles with a fine needle on the stage of a dissecting microscope. About 40-80 fully-grown GV-stage oocytes can be obtained from a single hormonally primed animal. In order to induce resumption of meiotic maturation, oocytes were washed out of IBMX by transferring them through sequential IBMX-free micro-drops of pre-warmed M16 medium (Sigma) under mineral oil (Sigma). Oocytes were then cultured in micro-drops of M16 medium under mineral oil at 37°C in a humidified atmosphere of 5% CO_2 in air.

Oocyte microinjection

GV-stage oocytes were microinjected in drops of pre-warmed IBMX-treated M2 medium under mineral oil as described previously (Gui and Homer, 2012; Homer et al., 2009). Holding and injection pipettes were made from sterile filament-free GC100T-10 glass (Clarke Electromedical Instruments, Berkshire, UK). Injection pipettes were made to have very fine calibre tips using pre-calibrated pipette pullers. A micro-forge was used for making holding pipettes and to give their distal ends (~2 mm long) an angle of approximately 50° to the main shaft. Microinjections were carried out on a Leica DM IRB (Leica Microsystems

UK Ltd., Milton Keynes, UK) inverted microscope equipped with Narishige hydraulic three-dimensional micromanipulators (Narishige Inc., Sea Cliff, NY, USA) using a 5×/0.12NA objective combined with a 1.5× magnifier. Injection pipettes were back-filled with about 1 μl of test solution. GV-stage oocytes were immobilised by applying suction through the holding pipette following which the tip of the injection pipette was introduced across the zona pellucida and oolemma into the ooplasm aided by a brief pulse of negative capacitance provided by an IE-251A Intracellular Electrometer (Warner Instruments LLC, Hamden, CT, USA). A pressure pulse was then applied to the test solution in the injection pipette using a PV820 Pneumatic PicoPump (World Precision Instruments, Sarasota, FL, USA) in order to introduce controlled volumes of test solution amounting to roughly 3%-5% of the oocyte volume (estimated to be 250 pl) as determined by cytoplasmic displacement. Following microinjection, oocytes were allowed to recover for 30-60 min in M2 medium. In order to induce GVBD, oocytes were then washed out of IBMX and transferred into M16 medium for longer term culture as described above.

Histone H1 kinase assays

Kinase assays were performed based on a previously described method (Kubiak et al., 1993). Groups of 15 oocytes were washed three times in 1% polyvinylpyrolidone (PVP) in PBS and lysed by repeated snap-freezing on dry-ice and thawing (x3) before storage at -80ºC until use. For each reaction, the lysed oocyte sample was mixed with twice the volume of 2× histone kinase buffer [80 mM β-glycerophosphate, 20 mM EGTA pH 7.3, 15 mM $MgCl_2$, 1 mM DTT and protease inhibitor cocktail (Roche)] and incubated with 3.3 mg/ml histone H1 (Sigma), 1 mM ATP and 0.25 mCi/µl [^{32}P]ATP for 50 minutes at 37ºC. The reaction was stopped by adding 4× SDS sample buffer (Invitrogen), heating for 5 minutes at 90ºC and cooling on crushed ice. Proteins were then resolved on 4-12% Bis-Tris gels (NuPAGE;

Invitrogen) after which the incorporation of [^{32}P] was analysed using a PhosphorImager (Molecular Dynamics).

Immunofluorescence

As described previously (Gui and Homer, 2012; Homer et al., 2009; Wassmann et al., 2003), oocytes were very briefly washed through PHEM solution (60 mM PIPES at pH 6.9, 25 mM HEPES, 10 mM EGTA, 2 mM $MgCl_2.7H_20$), before being pre-permeabilised in 0.25% Triton X-100 (Sigma) in PHEM for 5-10 seconds at room temperature (RT). Oocytes were then fixed in 3.7% paraformaldehyde (Sigma) in PHEM for 30 minutes before being permeabilised for 10 minutes in 0.25% Triton X-100 in PBS. After washing in PBS containing 0.5% BSA for 5 minutes at RT, non-specific binding sites were blocked by overnight incubation in PBS containing 3% BSA and 0.05% Tween-20 (blocking solution) at 4°C. The following morning, after being allowed to return to RT, oocytes were probed with primary antibodies.

For analysing cold-stable microtubules (see Figure S3), oocytes were incubated in ice-cold M2 medium for 10 min before being fixed in 3.7% paraformaldehyde in 100 mM PIPES at pH 6.8, 10 mM EGTA, 1 mM $MgCl_2$ and 0.2% Triton X-100 (Lampson and Kapoor, 2005). Oocytes were then blocked as described above before being probed with antibodies.

For immunolabelling, the following primary antibodies and dilutions were used as the first layer: 1:40 human ACA (ImmunoVision Inc., Springdale, AR, USA)(Duncan et al., 2009); 1:800 mouse anti-β-tubulin (Sigma) and 1:100 rabbit panHec1 antibody (Diaz-Rodríguez et al., 2008; a very kind gift from Robert Benezra, Memorial Sloan Kettering Cancer Center, USA). For the second layer, the following secondary antibodies were used: 1:200 of either Alexa Fluor 488- or Alexa Fluor 546-labelled goat anti-human (Invitrogen Ltd., Molecular Probes, Paisley, UK) for detecting ACA; 1:200 Alexa Fluor 633-labelled

goat anti mouse (Invitrogen) for detecting β-tubulin and 1:200 Alexa Fluor 546-labelled goat anti rabbit (Invitrogen) for detecting Hec1. DNA was labelled using Hoechst 33342 (1 µg/ml; B2261, bisBenzimide; Sigma) in PBS for 30-60 seconds at RT. Oocytes were then transferred to 1-2 µl micro-drops of PBS under mineral oil in glass bottom dishes for confocal imaging.

Image acquisition and analysis

Confocal microscopy

Confocal images were acquired with a Zeiss LSM 510 META (Carl Zeiss Imaging) equipped with a C-Apochromat 63×/1.2 NA water immersion objective and four lasers. Hoechst 33342 and DAPI were excited with a 364 nm UV laser and detected using a 385-470 nm band-pass emission filter; GFP and Alexa Fluor 488 were excited with the 488 nm line of an argon laser and detected using a 505-550 nm band-pass emission filter; Alexa Fluor 546 was excited using the 543 nm Helium/Neon1 laser and detected using a 560-615 nm band-pass emission filter, and Alexa Fluor 633 was excited using the 633 nm Helium/Neon2 laser and detected using a 650 nm long-pass emission filter. Confocal images were processed using Zeiss LSM Image Browser software and assembled into display panels using Adobe Photoshop (Adobe Systems Inc., San Jose, CA, USA).

Measuring spindle lengths and widths

It was important to consider that spindles can assume multiple orientations within the relatively large 3-dimensional spherical volume of the oocyte. In order to avoid errors due to parallax when determining spindle dimensions, we only analysed oocytes that were oriented in such a manner that the longest axis of their spindles were in the horizontal plane. Oocytes could be "rolled" using a glass pipette until their spindles were favourably orientated. Once oocytes were assessed to be in the correct orientation, spindle length was determined as the distance between two points located at the outermost extremities of the spindle and connected

by an axis that was parallel to the longest axis of the spindle. Spindle width was measured between two points at the outermost extremities of the spindle along an axis that was perpendicular to the longest spindle axis.

Immunoblotting

For sample collection, oocytes were washed in 1% polyvinylpyrolidone (PVP) in PBS, lysed in LDS sample buffer (NuPAGE; Invitrogen), snap-frozen and stored at -80°C. For blotting, samples were thawed on ice before adding reducing agent (NuPAGE; Invitrogen) and heated at 70°C for 10 minutes. Proteins were resolved on pre-cast 4-12% Bis-Tris gels (NuPAGE; Invitrogen) for 50 min at 200 V according to the manufacturer's recommendations. For detecting CENP-E, due to its relatively large size (287 kDa; see Figure S4A), 3-8% Tris-acetate gels (NuPAGE; Invitrogen) were used to separate proteins. Proteins were then transferred to PVDF membranes (Immobilon-P; Millipore) using the XL II Blot Module (Invitrogen). Following transfer, membranes were blocked for 1 h at RT in 3% BSA in TBS (25 mM Tris, 150mM NaCl, pH 8.0) containing 0.05% Tween-20 (TBST). Membranes were then incubated overnight at 4°C with primary antibody in blocking solution followed by either an HRP-conjugated goat anti-mouse or goat anti-rabbit antibody (Dako UK Ltd., Cambridgeshire, UK) as the second layer. The following primary antibodies and dilutions were used: 1:3000 rabbit panHec1 antibody; 1:400 mouse anti-cyclin B (ab72; Abcam); 1:200 mouse anti-Cdh1 (Abcam); 1:1000 mouse anti-securin (Abcam); 1:100 mouse monoclonal anti-CENP-E (Abcam), and; 1:400 mouse anti-actin (Abcam). As described previously (Gui and Homer, 2012; Homer et al., 2009), HRP-conjugated secondary antibodies were detected using the ECL Plus™ chemiluminescence detection system (GE Healthcare UK Ltd, Buckinghamshire, UK) and protein bands were semi-quantitatively

assayed using the ChemiDoc XRS Imaging System (Bio-Rad). Actin served as an internal control to ensure even sample loading and gel transfer.

Immunoprecipitation

Oocytes were washed in 1% PVP in PBS and re-suspended in 1ml of non-denaturing lysis buffer (50 mM Tris HCL pH 7.5, 50 mM NaCl, 1.0 % Triton X-100, complete mini protease inhibitor [Roche], 1 mM DTT). The lysate was centrifuged at 10,000 rpm for 10 min at 4°C and the supernatant was collected. For pre-clearing, 25 µl of protein G-coupled sepharose beads (GE Healthcare) was added to the supernatant and rotated for 1 h at 4°C and then centrifuged at 2000 rpm for 1 min. The resulting supernatant was incubated with either anti-Hec1 or anti-securin antibody for 1 h at 4°C following which, 100 µl of protein G-coupled sepharose beads was added and incubated for a further 1 h at 4°C. The beads were recovered by centrifugation and washed 4 times with non-denaturing lysis buffer. Finally, the immunoprecipitated proteins were eluted with SDS sample buffer by incubating for 3 min at 95 °C. Proteins were then resolved using SDS-PAGE and immunoblotted for securin, cyclin B2 or cyclin B1 as described above.

Morpholinos and cRNA constructs

Morpholinos were used for protein knockdown as described before (Gui and Homer, 2012; Homer et al., 2009; Homer et al., 2005b; Homer et al., 2005a). For mock depletion, we utilized a standard control morpholino (ControlMO). For depleting murine Hec1 (mHec1) in mouse oocytes, we used a morpholino designed to target *mHec1* (NM_023294) designated HecMO (5′-AGGTGGAAACTGAACTGCGCTTCAT-3′). For depleting murine cyclin B2 we used a morpholino designed to target *mCCNB2* (NM_007630; encoding cyclin B2) designated B2MO (5′-CCGCCCTGGCAAGTGCGGACGA-3′). Morpholinos designed

against *mCDH1* (Cdh1MO), *mMAD2* (Mad2MO) and *mCENP-E* (CENPEMO) have been described previously (Gui and Homer, 2012; Homer et al., 2009; Homer et al., 2005b; Homer et al., 2005a; Marangos et al., 2007; Marangos and Carroll, 2008; Reis et al., 2006; Reis et al., 2007). All morpholinos were supplied by Gene Tools (Gene Tools LLC, Philomath, OR, USA). GV-stage oocytes were microinjected with morpholinos, and maintained for 24 h in medium supplemented with IBMX so as to prevent GVBD thereby allowing time for protein knockdown.

For constructing cRNA encoding human Hec1 (hHec1), *hHec1* cDNA contained within the pCMV-SPORT6 vector (Clone ID: 5164424) was purchased from the IMAGE Consortium (Source BioScience Geneservice™, Cambridge, UK). For constructing cRNA encoding 9A-Hec1 – a mutant version of hHec1 in which all nine putative Aurora kinase target phosphorylation sites within the unstructured N-terminal tail domain have been mutated to Ala to prevent phosphorylation (DeLuca et al., 2011; Guimaraes et al., 2008; Sundin et al., 2011) – we used a plasmid containing *9A-Hec1-GFP* (a very kind gift from Dr Jennifer DeLuca, Colorado State University, USA). Sequences encoding *hHec1* and *9A-Hec1* were subcloned into the SP6 promoter-containing pCS2+ vector. For constructing cRNA encoding mcyclin B2 and mcyclin B2-GFP, we used T7 promoter-containing cDNAs (OriGene; MC203740 and MG206246). For generating the D-box-mutated mcyclin B2 construct, we mutated two residues in the D-box of mcyclin B2 – RAVLEEIGN to GAVSEEIGN – previously shown to confer stability on mcyclin B2 (Brandeis and Hunt, 1996), as we did previously for generating a KEN-box BubR1 mutant (Homer et al., 2009). cRNAs were made from linearised templates either by SP6 (*hHec1* and *9A-Hec1*) or T7 (*cyclin B2,* $^{\Delta D\text{-}box}$*cyclin B2* and *cyclin B2-GFP*) promoter-driven *in vitro* transcription using the mMESSAGE mMACHINE kit (Ambion, Applied Biosystems, Warrington, UK), polyadenylated, purified and dissolved in nuclease-free water for microinjection at a final

concentration of approximately 0.5-1 μg/μl as described previously (Homer et al., 2009; Homer et al., 2005b; Homer et al., 2005a).

Statistical Analysis

The mean, standard error of mean (SEM) and two-tailed Student's t-test were calculated using GraphPad Prism 5 (GraphPad software, CA, USA). p values < 0.05 were considered significant. Data are presented in graphs as mean ± SEM.

Supplemental References

Brandeis,M. and Hunt,T. (1996). The proteolysis of mitotic cyclins in mammalian cells persists from the end of mitosis until the onset of S phase. EMBO J 15, 5280-5289.

DeLuca,J., Dong,Y., Hergert,P., Strauss,J., Hickey,J., Salmon,E., and McEwen,B. (2005). Hec1 and nuf2 are core components of the kinetochore outer plate essential for organizing microtubule attachment sites. Mol Biol Cell 16, 519-531.

DeLuca,K.F., Lens,S.M., and Deluca,J.G. (2011). Temporal changes in Hec1 phosphorylation control kinetochore-microtubule attachment stability during mitosis. J Cell Sci 124, 622-634.

Diaz-Rodríguez,E., Sotillo,R., Schvartzman,J.M., and Benezra,R. (2008). Hec1 overexpression hyperactivates the mitotic checkpoint and induces tumor formation in vivo. Proc Natl Acad Sci U S A 105, 16719-16724.

Duncan,F.E., Chiang,T., Schultz,R.M., and Lampson,M.A. (2009). Evidence that a defective spindle assembly checkpoint is not the primary cause of maternal age-associated aneuploidy in mouse eggs. Biol Reprod 81, 768-776.

Gui,L. and Homer,H. (2012). Spindle assembly checkpoint signalling is uncoupled from chromosomal position in mouse oocytes. Development.

Guimaraes,G.J., Dong,Y., McEwen,B.F., and Deluca,J.G. (2008). Kinetochore-microtubule attachment relies on the disordered N-terminal tail domain of Hec1. Curr Biol 18, 1778-1784.

Homer,H., Gui,L., and Carroll,J. (2009). A spindle assembly checkpoint protein functions in prophase I arrest and prometaphase progression. Science 326, 991-994.

Homer,H., McDougall,A., Levasseur,M., Murdoch,A., and Herbert,M. (2005a). Mad2 is required for inhibiting securin and cyclin B degradation following spindle depolymerisation in meiosis I mouse oocytes. Reproduction 130, 829-843.

Homer,H., McDougall,A., Levasseur,M., Yallop,K., Murdoch,A., and Herbert,M. (2005b). Mad2 prevents aneuploidy and premature proteolysis of cyclin B and securin during meiosis I in mouse oocytes. Genes Dev 19, 202-207.

Kubiak,J., Weber,M., de Pennart,H., Winston,N., and Maro,B. (1993). The metaphase II arrest in mouse oocytes is controlled through microtubule-dependent destruction of cyclin B in the presence of CSF. EMBO J 12, 3773-3778.

Lampson,M. and Kapoor,T. (2005). The human mitotic checkpoint protein BubR1 regulates chromosome-spindle attachments. Nat Cell Biol 7, 93-98.

Marangos,P. and Carroll,J. (2008). Securin regulates entry into M-phase by modulating the stability of cyclin B. Nat Cell Biol 10, 445-451.

Marangos,P., Verschuren,E., Chen,R., Jackson,P., and Carroll,J. (2007). Prophase I arrest and progression to metaphase I in mouse oocytes are controlled by Emi1-dependent regulation of APCCdh1. J Cell Biol 176, 65-75.

Reis,A., Chang,H., Levasseur,M., and Jones,K. (2006). APCcdh1 activity in mouse oocytes prevents entry into the first meiotic division. Nat Cell Biol 8, 539-540.

Reis,A., Madgwick,S., Chang,H.Y., Nabti,I., Levasseur,M., and Jones,K.T. (2007). Prometaphase APCcdh1 activity prevents non-disjunction in mammalian oocytes. Nat Cell Biol 9, 1192-1198.

Sundin,L.J., Guimaraes,G.J., and Deluca,J.G. (2011). The NDC80 complex proteins Nuf2 and Hec1 make distinct contributions to kinetochore-microtubule attachment in mitosis. Mol Biol Cell 22, 759-768.

Wassmann,K., Niault,T., and Maro,B. (2003). Metaphase I arrest upon activation of the MAD2-dependent spindle checkpoint in mouse oocytes. Curr Biol 13, 1596-1608.
